# Causal Mediation Analysis with Multiple Mediators

**DOI:** 10.1111/biom.12248

**Published:** 2014-10-28

**Authors:** R M Daniel, B L De Stavola, S N Cousens, S Vansteelandt

**Affiliations:** 1 Centre for Statistical Methodology, London School of Hygiene and Tropical MedicineKeppel Street, London WC1E 7HT, UK; 2 Department of Applied Mathematics, Computer Science and Statistics, Ghent UniversityBelgium

**Keywords:** Causal pathways, Decomposition, Multiple mediation, Natural path-specific effects

## Abstract

In diverse fields of empirical research—including many in the biological sciences—attempts are made to decompose the effect of an exposure on an outcome into its effects via a number of different pathways. For example, we may wish to separate the effect of heavy alcohol consumption on systolic blood pressure (SBP) into effects via body mass index (BMI), via gamma-glutamyl transpeptidase (GGT), and via other pathways. Much progress has been made, mainly due to contributions from the field of causal inference, in understanding the precise nature of statistical estimands that capture such intuitive effects, the assumptions under which they can be identified, and statistical methods for doing so. These contributions have focused almost entirely on settings with a single mediator, or a set of mediators considered *en bloc*; in many applications, however, researchers attempt a much more ambitious decomposition into numerous path-specific effects through many mediators. In this article, we give counterfactual definitions of such path-specific estimands in settings with multiple mediators, when earlier mediators may affect later ones, showing that there are many ways in which decomposition can be done. We discuss the strong assumptions under which the effects are identified, suggesting a sensitivity analysis approach when a particular subset of the assumptions cannot be justified. These ideas are illustrated using data on alcohol consumption, SBP, BMI, and GGT from the Izhevsk Family Study. We aim to bridge the gap from “single mediator theory” to “multiple mediator practice,” highlighting the ambitious nature of this endeavor and giving practical suggestions on how to proceed.

## 1. Introduction

Exploring the relative strength of different pathways from an exposure to an outcome is a topic that has interested scientists across diverse fields for many decades. Early literature (Wright, 1921) through to the 1980s (Bentler, 1980; Baron and Kenny, 1986) focused on path analytic approaches, based on linear regression and structural equation models (SEMs). Under stringent parametric constraints, particular combinations of parameters from these models were taken to represent path-specific effects.

Starting with Robins and Greenland (1992), then Pearl (2001), followed by an explosion of recent contributions (see Ten Have and Joffe, 2012, and references therein, and more recent articles by VanderWeele and coauthors), the formal language and estimation methods from the field of causal inference have shone light on this problem and widened the scope of such analyses, under more explicit assumptions.

**Figure 1:**
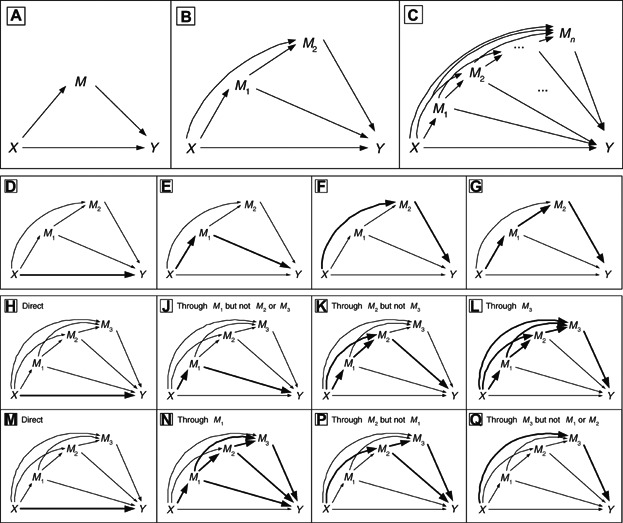
Top line: representations of mediation with (A) one, (B) two, and (C) *n* mediators, causally ordered. Second line: a depiction of mediation through two causally ordered mediators, with each of the four paths from *X* to *Y* highlighted; (D) shows the direct path (through neither 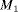 nor 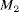), (E) the indirect path through 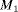 alone, (F) the indirect path through 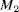 alone, and (G) the indirect path through both 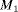 and 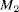. Lines 3 and 4: an illustration of the two possible ways of defining mediator-specific natural effects through three mediators. (H)–(L) show the first way and (M)–(Q) the second.

Robins and Greenland (1992) and Pearl (2001) used potential outcomes (Neyman, 1923; Rubin, 1978) to give model-free definitions of direct and indirect effect estimands. Informally, a direct effect acts around a mediating variable of interest, whereas the indirect effect acts through this mediator; “direct” thus refers to all other pathways other than through the mediator being considered. The mediator could be multivariate, but if so its constituent variables are considered *en bloc*: the direct effect acts around them all, and the indirect effect is through at least one of them without being further disentangled (Figure 1A).

In a setting with two mediators, 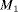 and 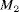 (see Figure1B), there are four possible pathways from exposure (*X*) to outcome (*Y*): through 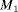 alone, through 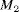 alone, through both and through neither. In this article, our primary aim is to express the total causal effect of *X* on *Y* as the sum of separate effects along each of the possible pathways: the *finest possible decomposition*. The existing literature on multiple (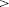
2) pathways from exposure to outcome can be characterized as follows; either (1) 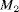 is the mediator of interest, and 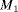 is treated as a mediator–outcome confounder affected by exposure, leading to a coarser two-way decomposition into an effect (indirect) through 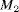 and an effect (direct) not through 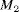 (Tchetgen Tchetgen and Shpitser, 2012; Vansteelandt and VanderWeele, 2013; VanderWeele and Chiba, 2014; VanderWeele, Vansteelandt, and Robins, 2014), (2) path-specific effects are estimated, but not in such a way that their sum equals the total causal effect (Avin, Shpitser, and Pearl, 2005; Albert and Nelson, 2011), and (3) the multiple mediators do not causally affect one another (MacKinnon, 2000; Preacher and Hayes, 2008), that is, the arrow from 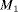 to 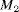 in Figure 1B is assumed absent, reducing the number of path-specific effects to three. Imai and Yamamoto (2013) fall into all three categories in different sections of their article, but at no point discuss the finest possible decomposition of the total causal effect in the presence of the arrow from 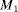 to 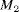.

The outline for the remainder of the article is as follows. In Section 2 we briefly review mediation estimands in the single mediator setting. In Section 3 we give our proposed classification of estimands when there are two causally ordered mediators, showing how decomposition can be achieved, and suggesting strategies for reducing complexity. Section 4 gives sufficient assumptions under which the estimands introduced in Section 3 can be identified, including details of a sensitivity analysis, and estimation methods are discussed briefly in Section 5. The approach is illustrated in Section 6 using data from the Izhevsk Family Study, and we conclude with some discursive remarks in Section 7. Extensions to *n* causally ordered mediators (Figure 1C) are given in the Web Appendix.

## 2. A Brief Review of Causal Mediation Estimands for One Mediator

We briefly review mediation estimands for a single mediator. A more detailed account is given in Daniel et al. (2014).

Consider an exposure *X*, mediator *M* and outcome *Y* (Figure 1A). The total, direct and indirect effects defined by Robins, Mark, and Newey (1992) and Pearl (2001) involve the counterfactual variables 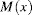, 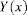, 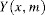, and 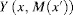. These are, respectively, the value *M* would take were *X* set to *x*, the value *Y* would take were *X* set to *x*, the value *Y* would take were *X* set to *x* and *M* to *m*, and the value *Y* would take were *X* set to *x* and *M* to 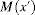.

For simplicity, we take *X* to be binary. The *controlled direct effect* (CDE) at level *m* of *M* is 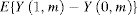, the *pure natural direct effect* (PNDE) is 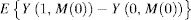, and the *total natural direct effect* (TNDE) is 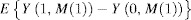. In each definition, *M* takes the same value in both halves of the contrast, corresponding to a “direct” effect. For the CDE, this value of *M* is the same for all individuals, whereas for the natural direct effects, it differs by individual, according to the value that *M* would naturally take were *X* set to 0 (pure) or 1 (total).

The *pure natural indirect effect* (PNIE) is 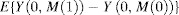 and the *total natural indirect effect* (TNIE) is 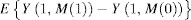. Note that these correspond to the idea of an indirect (mediated) effect, since they capture the effect on *Y* of changing *X*, but only via its effect on *M*. The first argument of the counterfactual is the same in both halves of each contrast, but this fixed value can be either 0 (pure) or 1 (total).

Note that the sum of the PNDE and TNIE and the sum of the TNDE and PNIE are the same, and that this quantity is the *total causal effect* (TCE) of *X* on *Y*: 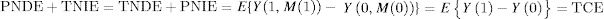. That is, there are two definitions (pure and total) of natural direct and indirect effects, and two ways of decomposing the TCE into a sum of a natural direct and indirect effect. VanderWeele (2013) shows that the difference 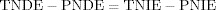 corresponds to a “mediated interaction,” non-zero if and only if there is an effect of *X* on *M* and an interaction between *X* and *M* in their effect on *Y*. Thus the choice between the definitions/decompositions, which (in many contexts) is somewhat arbitrary, amounts to assigning the mediated interaction either to the direct or indirect effect.

## 3. Causal Mediation Estimands with Two Causally Ordered Mediators

Turning to the setting with two mediators (Figure 1B) we first note that 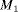 can affect 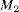 but not vice versa; in some applications, there may be doubt as to the direction of the arrow between 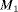 and 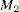, which would introduce further difficulties beyond the scope of this article. We define four *path-specific effects*—one not mediated by either 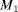 or 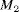 (Figure 1D), one through 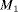 alone (Figure 1E), one through 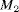 alone (Figure 1F), and one through both 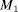 and 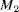 (Figure 1G)—such that these sum to the TCE.

### 3.1. Potential Values of Mediators and Outcome

Let 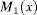, 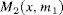, 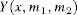, 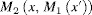, and 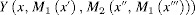 be defined according to the obvious extensions of the definitions given in Section A Brief Review of Causal Mediation Estimands for One Mediator.

### 3.2. Natural Direct Effects

Let the *natural-*000 *direct effect* through neither 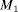 nor 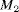 be 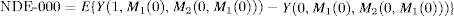. This is the obvious extension of the PNDE to two mediators and is the direct effect defined by Avin et al. (2005) and Albert and Nelson (2011). The first argument is the only one that changes, from 1 to 0, making it a direct effect. The other three arguments are fixed at 0; this is why we label it “000.” Rather than two types of effect (pure and total), there are now 8 types of effect—000, 100, 010, 001, 110, 101, 011, and 111—corresponding to each of the ways in which the other three arguments could be set. See Table 1 for all 8 definitions.

**Table 1:** The top half of this table gives the definitions of all natural path-specific effects when there are two causally ordered mediators. There are eight versions (one level-*0*, three level-*1*, three level-*2*, and one level-*3*) of each of the four effects (direct, indirect through 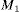 alone, indirect through 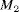 alone, and indirect through both 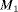 and 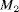). The ones shown in bold are the ones defined in Sections Natural Direct Effects and Indirect Effects that Allow Decomposition. Note that the level-*0* effects are those studied by Avin et al. (*2005*) and Albert and Nelson (*2011*). The bottom half of the table gives the definitions of the mediator-specific effects introduced in Section Mediator-specific natural effects

Path	Level	Effect	Definition
	0	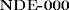	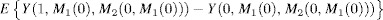
	1	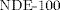	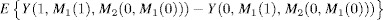
	1	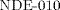	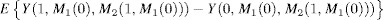
Direct	1	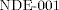	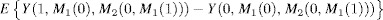
(through 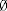 )	2	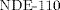	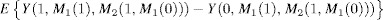
	2	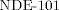	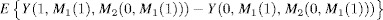
	2	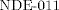	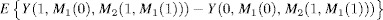
	3	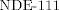	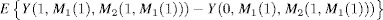
	0	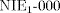	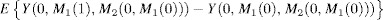
	1	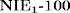	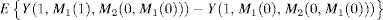
Indirect	1	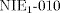	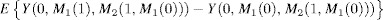
through	1	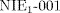	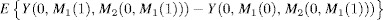
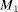	2	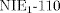	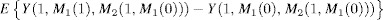
only	2	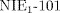	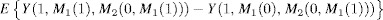
	2	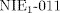	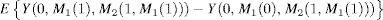
	3	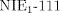	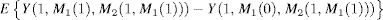
	0	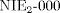	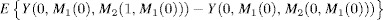
	1	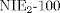	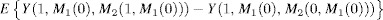
Indirect	1	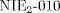	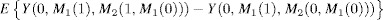
through	1	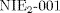	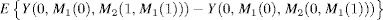
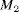	2	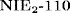	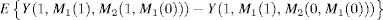
only	2	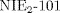	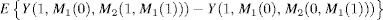
	2	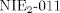	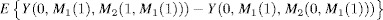
	3	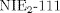	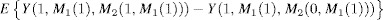
	0	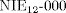	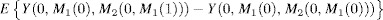
	1	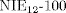	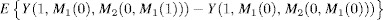
Indirect	1	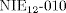	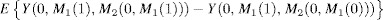
through	1	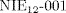	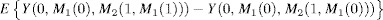
both 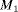	2	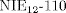	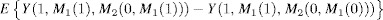
and 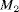	2	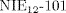	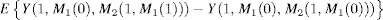
	2	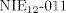	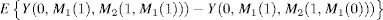
	3	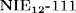	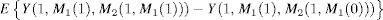
		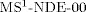	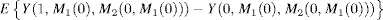
		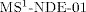	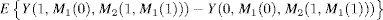
		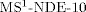	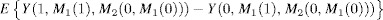
		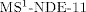	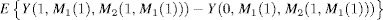
		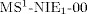	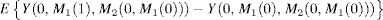
		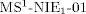	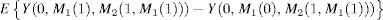
		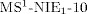	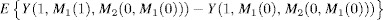
		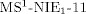	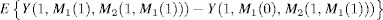
		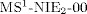	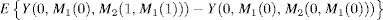
		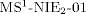	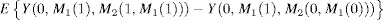
		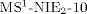	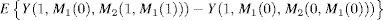
		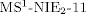	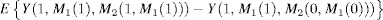
		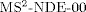	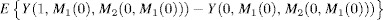
		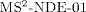	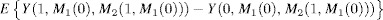
		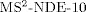	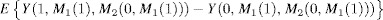
		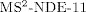	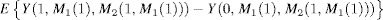
		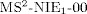	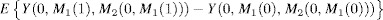
		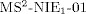	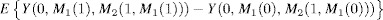
		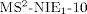	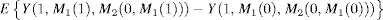
		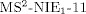	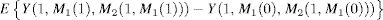
		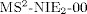	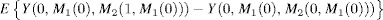
		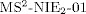	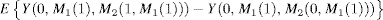
		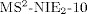	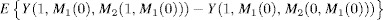
		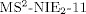	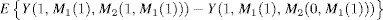

### 3.3. Indirect Effects that Allow Decomposition

We now define indirect effects through 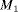 alone, 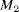 alone, and through both 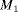 and 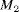 such that their sum, together with the natural-000 direct effect, is equal to the TCE.

The *natural-*100 *indirect effect through* 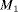 *alone* is 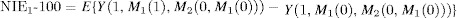. Intuitively, this corresponds to an indirect effect of *X* on *Y* via 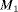 alone since it captures the effect of *X* on *Y* only through its effect on 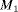, with the effect of 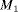 on 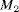 removed. The argument that differs between the two potential outcomes is the second one, the *x* shown here: 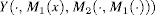. The first argument is set to 1 in both potential outcomes, whereas the arguments that follow *x* are set to 0; this is why we label it “100.” Similarly, the *natural-110 indirect effect through* 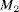 *alone* is 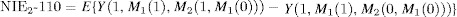, and the *natural-111 indirect effect through both* 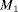 *and* 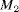 is 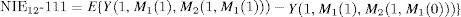, with each of the seven other types given in Table 1. Note that only the 000 effects have been defined in previous literature (Avin et al., 2005; Albert and Nelson, 2011).

For each effect type, we define its *level* to be the sum of the three fixed *x*-arguments. Thus NDE-000 is a level-0 effect, NIE_1_ -100 is a level-1 effect, etc.

Using the effects chosen above, it is easily verified that the total causal effect decomposes:


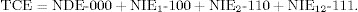
(1)

Note that Albert and Nelson (2011) study 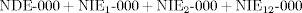, and calculate each path-specific 000 effect as a proportion of this sum. Since this sum is *not* in general equal to the total causal effect, these proportions are not analogous to the “proportion mediated” typically calculated in settings with one mediator (Pearl, 2001).

### 3.4. Alternative Decompositions

The decomposition given in (1) is not the only such decomposition. With one mediator there are two types (pure and total) of two path-specific effects (direct and indirect); with two mediators, there are eight types of four path-specific effects. Forming sums by choosing one type of each effect, with one mediator, we found that two out of the four possible sums equate to the TCE (PNDE+TNIE=TNDE+PNIE=TCE, but PNDE+PNIE
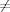
TCE and TNDE+TNIE
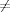
TCE). With two mediators, there are 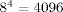 possible sums, and 24 of them equate to the TCE. That is, there are exactly 24 ways of decomposing the TCE into a sum of its path-specific components through and around two mediators: the decomposition shown in (1) and 23 others (see Table 2). That these 24 are unique and represent all possible decompositions follows from the more general argument (for *n* mediators) given in Web Appendix A, where we show that there are 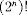 ways of decomposing a TCE into a sum of path-specific effects through *n* mediators.

**Table 2:** All *24* possible decompositions of the total causal effect (TCE) into a direct effect (NDE), an indirect effect via 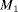 alone (NIE_1_), an indirect effect via 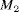 alone (NIE_2_), and an indirect effect via both 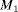 and 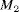 (NIE_12_). In each decomposition, there is one level-*0* effect, one level-*1* effect, one level-*2* effect, and one level-*3* effect. The definitions of each of these effects is given in Table 1. In columns *2–5*, the effect types are labeled: *1=000*, *2=100*, *3=010*, *4=001*, *5=110*, *6=101*, *7=011*, and *8=111*

	Effect and type				
Decomposition	NDE	NIE_1_	NIE_2_	NIE_12_	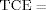
1	1	2	5	8	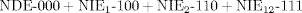
2	1	2	8	5	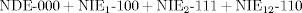
3	1	5	2	8	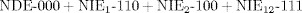
4	1	6	8	2	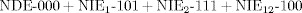
5	1	8	2	6	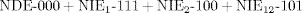
6	1	8	6	2	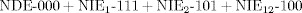
7	2	1	5	8	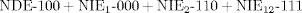
8	2	1	8	5	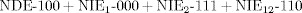
9	3	5	1	8	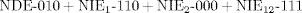
10	3	8	1	6	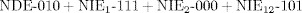
11	4	6	8	1	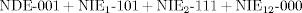
12	4	8	6	1	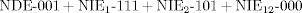
13	5	1	3	8	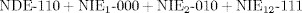
14	5	3	1	8	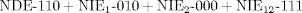
15	6	1	8	3	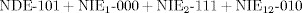
16	6	4	8	1	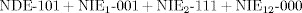
17	7	8	1	4	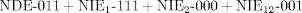
18	7	8	4	1	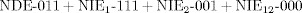
19	8	1	3	7	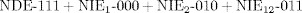
20	8	1	7	3	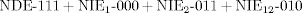
21	8	3	1	7	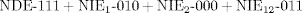
22	8	4	7	1	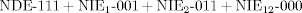
23	8	7	1	4	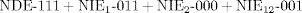
24	8	7	4	1	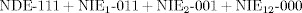

With 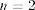, each decomposition includes one level-0, one level-1, one level-2, and one level-3 effect. In short, there are 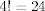 ways of allocating these four levels to the four paths, and this gives rise to the 24 possible decompositions.

### 3.5. Example: Linear Structural Equation Model with Interactions

For illustration, we suppose that the data were generated from a linear structural equation model with interactions (and, for simplicity, no confounders), that is, a model implying the following conditional expectations: 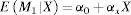, 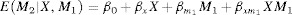 and 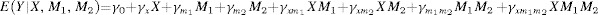. Note that once interaction terms (or other nonlinearities) are included in the SEM, the simple method of multiplying path coefficients to calculate path-specific effects cannot be applied (VanderWeele and Vansteelandt, 2009).

In Web Appendix B we derive each of the 32 path-specific estimands in this special case in terms of the parameters above, together with certain conditional variance/covariance terms. For example, we have that


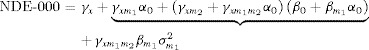


where 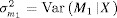, and


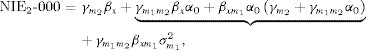


where the terms denoted by the underbraces could be set to zero by adding appropriate constants to 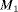 and 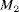 (so that 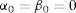); although in the presence of interactions these terms differ for different effect types (see Web Appendix B). Note that 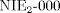, for example, contains 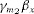, the term that would result from applying the “product of coefficients” methods to a linear model without interactions (Wright, 1921). It also has a further term involving 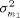 if there are two interactions present. A similar expression is seen for 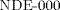, where the “standard” direct effect (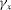) appears along with a variance term. The formulæ for some of the other effects involves the covariance of 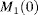 and 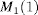; we return to this point later. Note that the natural effects derived here would coincide with those used in the LSEM approach in the absence of all interactions.

### 3.6. Practical Suggestions for Reducing Complexity

With two mediators, it can be feasible to estimate all 32 path-specific effects, and hence all 24 decompositions, and compare them. However, with more mediators, the complexity grows at such a rate that this becomes impractical, even for three mediators (see Web Appendix A). In this section, we give three suggestions for reducing this complexity.

#### 3.6.1. Focusing on effects of greatest substantive interest

Depending on the exposure, it can often be argued that the 000 effects are substantively most interesting, and easiest to interpret. For example, if 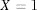 denotes a new experimental medical treatment, with 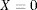 for the standard treatment, then the 000 effects are most naturally interpreted, since they entail setting the free arguments in the effect to what they would be under the standard treatment. If, in addition, one particular mediator is of greater interest than the others, then the number of decompositions one needs to consider could be partially reduced by focusing only on decompositions that include level 000 of the indirect effect through the mediator of interest (e.g., for 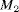, decompositions 9, 10, 14, 17, 21, and 23 in Table 2). With two mediators, therefore, this strategy reduces the number of decompositions from 24 to 6.

#### 3.6.2. Summary natural path-specific effects

We define the *summary natural path-specific effects* SNDE (direct), SNIE_1_ (through 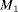 only), SNIE_2_ (through 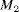 only) and SNIE_12_ (through both 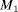 and 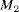) as follows:


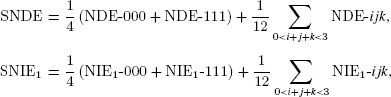


and similarly for 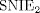 and 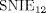.

The weights (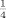 and 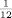) follow from how the path-specific types contribute to each of the 24 decompositions: in columns 2–5 of Table 2, each type-1 and type-8 (000 and 111) effect appears 6 times, and each of the other effect types appears twice. It follows therefore that


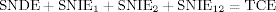
(2)

and (2) represents a summary of the 24 decompositions, which itself is a decomposition of the TCE into four (summary) path-specific effects. Whereas with one mediator, the summary direct and indirect effects can be interpreted as the direct and indirect effects that would be seen in a particular randomized experiment (see Web Appendix C), we are not aware of a similar intuitive interpretation of the summary effects for two or more mediators.

When summarizing the effects in this fashion, it would be useful also to consider the variability of the component effects, so that this information is not entirely lost. For example, for the direct effects, we define:


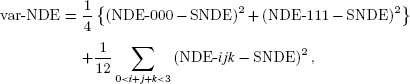


weighted to reflect that the SNDE will be closer to NDE-000 and NDE-111 than to the other effects. Similar expressions for var-NIE_1_, var-NIE_2_ and var-NIE_12_ are omitted.

#### 3.6.3. Mediator-specific natural effects

Another option is to focus on a coarser decomposition. Indeed, as the number of mediators increases, we are unlikely to be interested in each of the 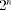 path-specific effects. For example, with two mediators, we could combine the effect through both 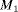 and 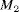 with either the effect through 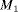 alone, or with the effect through 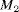 alone, leaving us with a decomposition into only three effects: the direct effect, and two *mediator-specific* effects. Graphically, the path shown in Figure 1G could either be combined with that of Figure 1F or with that of Figure 1E. Both lead to natural nested interpretations as follows. In the former (combining G and F, which we will denote as MS^1^, mediator-specific type 1) the mediator-specific direct effect is the effect through neither 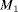 nor 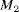, the mediator-specific effect through 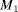 is the effect through 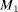 but not through 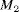, and the mediator-specific effect through 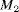 is all of the effect through 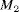. Similar definitions would apply to the latter (combining G and E, which we will denote as MS^2^). It is perhaps easier to understand this “nesting” argument, by generalizing to three mediators, as shown in Figure 1H–Q.

The algebraic definitions are given in the bottom half of Table 1. Note that such a sequential treatment of multiple mediators is also discussed in VanderWeele and Vansteelandt (2014).

These summaries do not assume no exposure–mediator or no mediator–mediator interactions, as would be required in linear structural equation modeling (see Web Appendix D). Discrepancies between these and estimates obtained under a no-interactions assumption would prompt more closely studying the original contributing path-specific effects.

## 4. Assumptions That Permit Identification

### 4.1. Identification Assumptions

Sufficient assumptions for the identification of the TCE are:

(T.1) *Consistency of X on Y*: 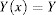 if 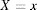. For those with exposure *x*, outcome *Y* and potential outcome 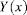 coincide (Rubin, 1978; Cole and Frangakis, 2009).
(T.2) *No unmeasured confounding of the X–Y relationship*: Formally, 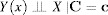 for all (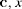), where 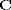 is a set of measured background confounders, not affected by *X*.

Assumption (T.1) is required for the TCE to be interpretable as the effect that would be seen in a hypothetical experiment in which we intervene on *X* in a well-defined fashion. The consistency assumption then states that the results are relevant for any kind of intervention which is such that it would have produced the data we have for those for whom 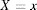 is naturally observed.

Assumption (T.2) states that, after taking into account observed background confounders 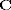, any remaining association between *X* and *Y* can be given a causal interpretation.

This intuition carries through to the extensions of these assumptions in the remainder of this section.

For the CDE, a sufficient set of assumptions is:

(C.1) *Consistency of* 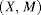 *on Y*: 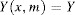 if 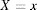 and 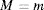.
(C.2) *No unmeasured confounding of the* 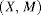
*–Y relationship*: 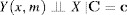 for all 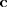 and 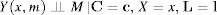 for all 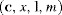, where 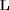 is a set of measured intermediate confounders, where “intermediate” is used to denote that 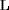 may be affected by *X* (but not by *M*).

If we assume that the data are generated from a non-parametric structural equation model (NPSEM, see Pearl, 2009; Daniel et al., 2014) then, for the identification of the PNDE, TNDE, PNIE and TNIE, a sufficient set of assumptions is (C.1), (C.2), and, in addition:

(N.3) *Consistency of X on M*: 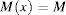 if 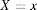.
(N.4) *No unmeasured confounding of the X–M relationship*: 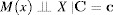 for all 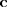.
(N.5) *No mediator–outcome confounders affected by X*, that is, no intermediate confounders 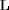.

Without the NPSEM assumption, (N.5) is replaced by 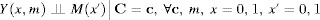, which is more difficult to interpret. Either version of assumption (N.5) can be relaxed, but only under strong parametric restrictions. For further details of all aspects of this subsection, see Daniel et al. (2014).

### 4.2. Assumptions for Identifying Path-Specific Effects with Two Causally Ordered Mediators

#### 4.2.1. Non-parametric identification

For the CDE with two mediators (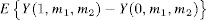), (C.1) and (C.2) generalize to:

(MC.1) *Consistency of* 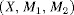 *on Y*.
(MC.2) *No unmeasured confounding of the* 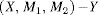 *relationship*: 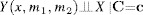 for all (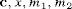), 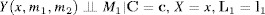 for all 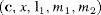 and 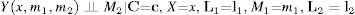 for all 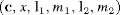, where 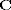 are measured background confounders (unaffected by *X*, 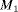 or 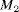), 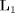 is a set of measured intermediate confounders, which may be affected by *X*, but not by 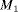, and 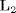 is a second set of measured intermediate confounders, which may be affected by *X* and/or 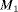, but not by 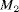. See Web Figure 5A.

Under (MC.1) and (MC.2), the CDE is then identified using the *g*-computation formula (Robins, 1986); see Web Appendix E.

The generalizations of (N.3)–(N.5) (for the natural effects) to two mediators, under the assumption that the data are generated from a NPSEM, are as follows:

(MN.3) *Consistency of X on* 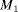 *and of* 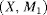 *on* 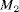.
(MN.4) *No unmeasured confounding of the X–*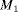 *or* 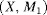
*–*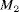 *relationships*: 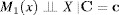 for all (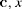), 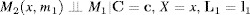 for all 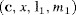 and 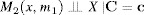 for all (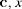).
(MN.5) *No mediator–outcome confounder affected by X*, that is, no 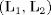 (Web Appendix F).

Each half of each of the natural path-specific effects in Table 1 is of the form


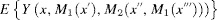
(3)

and thus if we could identify (3) under assumptions (MC.1), (MC.2) and (MN.3)–(MN.5), all effects in Table 1 would be identified. To this end, we have the following result:

Theorem 1. Under assumptions (MC.*1*), (MC.*2*) and (MN.*3*)–(MN.*5*), we have that:


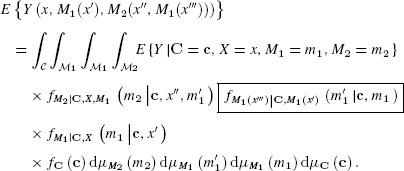
(4)

For the proof, see Web Appendix H.

Note that (4) involves one density (shown in a box) not written as a function of the distribution of the observed data. A sensitivity analysis when this is unknown is discussed in the next section. There are two special cases in which the boxed quantity in (4) is not required, or is trivially known.

**Special case 1**: 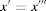


If 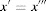, then 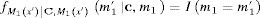. Thus all path-specific estimands in which 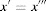 in *both* halves of the expression are nonparametrically identified under assumptions (MC.1), (MC.2), and (MN.3)–(MN.5). These are: NDE-000, NDE-010, NDE-101, NDE-111, NIE_2_ -000, NIE_2_ -100, NIE_2_ -011, and NIE_2_ -111. Also, note that MS^1^ -NDE-00 and MS^1^ -NDE-11, together with all of the MS^2^ -NDE, MS^2^ -NIE_1_ and MS^2^ -NIE_2_ effects, are made up of effects in which 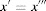, and thus are also identified under assumptions (MC.1), (MC.2) and (MN.3)–(MN.5).

**Special case 2**: No effect of 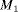 on 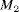


If there is no effect of 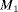 on 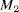, the calculation above simplifies as follows

Corollary 1. Under assumptions (MC.*1*), (MC.*2*), and (MN.*3*)–(MN.*5*), if there is no effect of 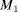 on 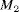:


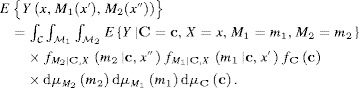


All effects (when 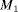 does not affect 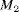) are thus nonparametrically identified under assumptions (MC.1), (MC.2), and (MN.3)–(MN.5).

In the absence of an effect of 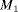 on 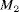, the definitions and decompositions given in Section Causal Mediation Estimands with Two Causally Ordered Mediators simplify. There is no longer a path through both 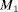 and 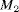, and thus the fourth argument in each half of each effect disappears. This leaves 12 effects, and 6 decompositions; the effects are listed in Table 3, with the decompositions given in Web Table 1. Some of these effects and decompositions correspond to those given by Imai and Yamamoto (2013); in particular, Imai and Yamamoto define the NDE-00, NDE-01, NDE-10, NDE-11, NIE_1_-00, NIE_1_-11, NIE_2_-00, and NIE_2_-11, but not the remaining 4 effects (see Table 3) and they point out that 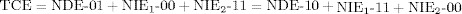 but do not give the other four possible decompositions (see Web Table 1). A summary of the comparison between the estimands defined and identified in the current manuscript versus those defined and identified in the previous literature is given in Web Table 2.

**Table 3:** The definitions of all natural path-specific effects when there are two mediators that are not causally ordered. There are four versions (one level-*0*, two level-*1*, and one level-*2*) of each of the three effects (direct, indirect through 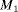, and indirect through 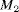; note that there is no effect through both 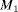 and 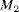 when the mediators are not causally ordered)

Path	Level	Effect	Definition
	0	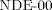	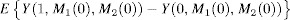
Direct	1	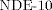	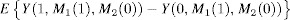
(through 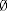 )	1	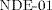	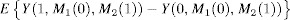
	2	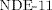	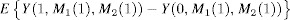
	0	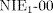	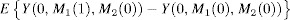
Indirect	1	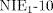	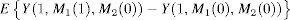
through 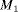	1	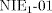	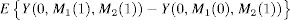
	2	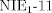	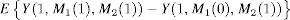
	0	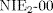	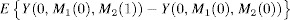
Indirect	1	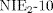	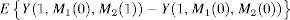
through 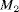	1	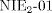	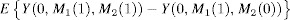
	2	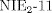	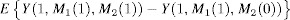

Note that the decompositions given in Web Table 1 apply also to the mediator-specific natural effects defined in Section Mediator-specific natural effects.

As already noted, Avin et al. (2005) define only 000 effects, but, by symmetry, their identification result applies also to the 111 effects. Insofar as they can be compared, our result agrees with that of Avin et al. since they conclude that the effect along the direct pathway (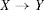) and the effect along the indirect pathway through 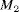 alone (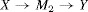) are identifiable, but that the effects along the other two pathways (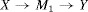 and 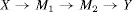) are not. This corresponds to what we find, since NDE-000, NDE-111, NIE_2_ -000, and NIE_2_ -111 are all included in our list of effects which can be estimated without the sensitivity parameter, whereas none of the NIE_1_ or NIE_12_ effects is included in this list.

#### 4.2.2. Identification and sensitivity analysis under a particular parametric model

When there is an effect of 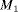 on 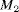, the effects not listed under “special case 1” above require knowledge of the boxed quantity in (4) when 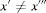. Under most estimation strategies (see Section A Note on Estimation Methods), we would assume a parametric model for the distribution of 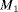 given *X* and 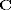, for example that 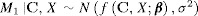, and we would estimate the parameters 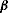 and 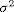 from data on 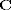, *X* and 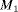. Under assumptions (MN.3) and (MN.4) and if our model for 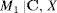 is correctly specified, this gives us 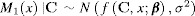 for 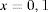. In this case, in order to know the boxed quantity in (4), we would need, in addition to this model, the conditional correlation between 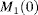 and 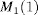 given 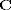. There is no information in the data on the value of this correlation; a sensible approach would thus be to vary this parameter in a sensitivity analysis.

For example, consider the following form for the SEM for 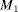: 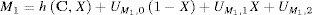, for some function 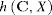, where 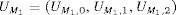 and


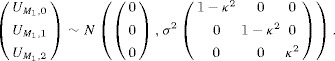


Then 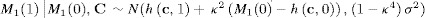. Note that 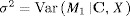 can be estimated from the data. However, the data contain no information on 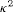, the proportion of residual variance shared across worlds; this becomes the sensitivity parameter, to be varied from 0 to 1. For more details, see Web Appendix J. An example of this sort of sensitivity analysis is given in Section An Illustrative Data Example: The Izhevsk Family Study.

A similar approach was taken by Daniels et al. (2012), for discrete mediators by Albert and Nelson (2011), and in the context of treatment noncompliance by Roy, Hogan, and Marcus (2008). Note that this sensitivity analysis solely assesses sensitivity to the arbitrary choice of conditional distribution of 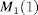 given 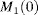 and 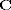; it does not explore sensitivity to departures from the other assumptions, namely (MC.1), (MC.2), and (MN.3)–(MN.5). An extensive literature on sensitivity analyses with respect to the single mediator versions of these assumptions exists, including in the presence of mediator–outcome confounders affected by the exposure (see, e.g., Imai, Keele, and Yamamoto, 2010; Tchetgen Tchetgen and Shpitser, 2012; VanderWeele and Chiba, 2014). In future work, we will extend these sorts of sensitivity analyses to the current setting.

An alternative route to parametric identification and sensitivity analysis would be to extend the “no interaction” assumption made by Robins and Greenland (1992) and relaxed by Imai and Yamamoto (2013). Given, however, that the 24 possible decompositions differ precisely when interactions are present, assuming them away may not be as attractive.

In Web Appendix K, we show what our identification results imply for the special case of the linear model with interactions introduced in Section Example: Linear Structural Equation Model with Interactions, and in Web Appendix L, we show how identification is achieved, up to a set of sensitivity parameters, in the presence of a restricted pattern of intermediate confounding.

## 5. A Note on Estimation Methods

The most obvious estimation approach is to posit parametric (regression) models for each density/expectation in the identifying equations above, to estimate their parameters from the observed data (e.g., by maximum likelihood), and then to evaluate the integrals analytically. Pearl (2009) calls this approach the *mediation formula*. Closely related to the *g-computation formula* (Robins, 1986), which can be used to estimate controlled direct effects in the presence of intermediate confounding, the mediation formula makes the additional step of integrating over the (conditional counterfactual) mediator distribution, in order to obtain natural effects (VanderWeele and Vansteelandt, 2009, 2010). When the integration is too cumbersome to be done analytically, it can instead be done by Monte Carlo simulation (Robins, 1986; Imai, Keele, and Tingley, 2010; Daniel, De Stavola, and Cousens, 2011).

The advantage of relying heavily on parametric models is that this approach is efficient when all models are correct; however, as pointed out by Robins and Wasserman (1997) and further discussed by Vansteelandt, Bekaert, and Lange (2012), the disadvantage is that it can be essentially impossible to specify these models such that they imply a sensible parsimonious model for the direct effect of interest. For this reason, and, more generally, to reduce reliance on parametric modelling assumptions, alternative semiparametric estimation approaches have been suggested (van der Laan and 2008; VanderWeele, 2009; Tchetgen Tchetgen and Shpitser, 2012; Vansteelandt et al., 2012; Zheng and van der Laan, 2012). G-computation has nevertheless turned out to be rather successful in recent empirical applications (Young et al., 2011; Westreich et al., 2012).

We therefore adopt the fully parametric approach, implemented by Monte Carlo simulation, extending it to handle multiple mediators and incorporating the sensitivity analysis of Section Identification and sensitivity analysis under a particular parametric model. In future work, semiparametric estimation methods will be explored.

## 6. An Illustrative Data Example: The Izhevsk Family Study

### 6.1. Data and Question of Interest

The population-based controls from a case-control study conducted in Izhevsk, Russia (Leon et al., 2007) are used to study the effect of heavy drinking during the previous year (defined as the consumption of 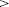
10 L ethanol) on systolic blood pressure (SBP), measured in mmHg. We decompose this into an effects via body mass index (BMI), via gamma-glutamyl transpeptidase (GGT), via both BMI and GGT, and a direct effect, that is, via other pathways. BMI is known to affect GGT (and not vice versa), and thus the set-up is as we have discussed, with 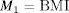 and 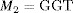. We estimate the path-specific effects using data on 1275 men with complete information on yearly ethanol consumption (from which “heavy drinking” is derived) and all baseline confounders: age (treated as a continuous variable), socio-economic status (SES) score (the first principal component from an asset score analysis), smoking status (current/ex/never), and cigarettes per day (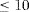,10–20,
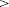
20): together we label these confounders 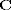 (Leon et al., 2007). Note that in this setting there are no (measured) intermediate confounders. Subjects with missing values of BMI, GGT and/or SBP are not excluded, since these partially observed records can be incorporated, under the missing at random assumption (Rubin, 1976). Some descriptive statistics are shown in Web Table 3.

### 6.2. Estimation by Parametric *G*-computation via Monte Carlo Simulation

Flexible parametric models for 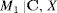, 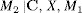, and 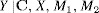 were explored. To render the normality assumption for the errors more tenable, 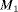 and 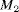 (i.e., BMI and GGT) were log-transformed. All models included all possible two- and three-way interactions between exposure and mediators, so that the path-specific effects of different types differ as much as the data dictate. In addition, quadratic terms for the continuous variables (age, SES, BMI, and GGT) were considered where relevant, as well as interactions between exposure and confounders; these were included only if they improved the AIC (see Web Appendix M).

Write 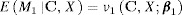, 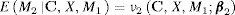, and 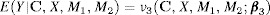 for the conditional expectations implied by this model, and let the error variances be 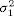, 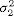 and 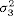, respectively.

The estimation of path-specific effects is carried out as follows.

1. Estimate the parameters (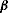,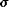) by OLS/ML.
2. For each subject *i*, draw 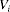 from 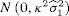. 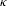 is the sensitivity parameter (see Web Appendix J), to be varied between 0 (no cross-world correlation conditional on 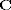) and 1 (perfect cross-world correlation conditional on 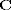).
3. For 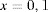, draw 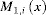 for each *i* from 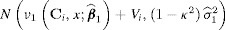.
4. For 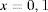 and 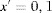, draw 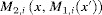 from 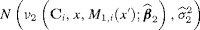.
5. For 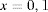, 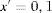, 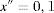 and 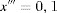, draw 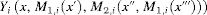 from 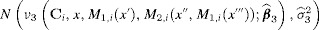.
6. To estimate each of the 32 effects 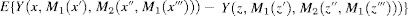, the empirical average across all subjects of 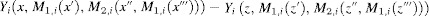 is found.

To decrease Monte Carlo error, the simulation is done on a dataset 1000 times the size of the original (with the values of 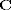 copied 1000 times), although the estimation of the parameters 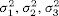 is based on the original sample. Standard errors are computed using the nonparametric bootstrap. For comparison, a LSEM (with no interactions) is also fitted.

### 6.3. Results

**Figure 2:**
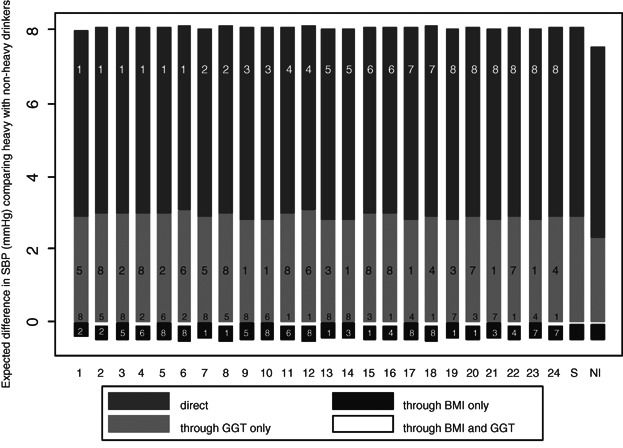
With 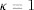 (perfect correlation between 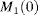 and 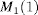 given 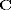), all 24 possible decompositions of the total causal effect of heavy drinking on SBP into four path-specific components: a direct effect unmediated by BMI or GGT, an indirect effect via BMI alone, an indirect effect via GGT alone, and an indirect effect via both BMI and GGT. The numbers superimposed on the bars represent the code for that effect type (as defined in the caption of Table 2). The numbers along the *x*-axis represent the decomposition number, also defined in Table 2.

The results are shown in Tables 4 and 5 and Web Figures 1 and 2. There is evidence of a total effect of heavy drinking on SBP, but the associated confidence interval is wide (mean difference 7.63 mmHg, 95% CI 3.89–11.37). Only a small proportion (1.7%) of the large variation in SBP across this sample of men is explained by the dichotomous heavy drinking variable. It is not surprising therefore that the estimates of the various path-specific effects are also imprecise. Examination of the residual distribution for each contributing associational model shows good agreement with the assumption of normality while evidence for the interaction terms was weak (see Web Table 5). There is evidence of a small indirect effect through GGT alone (mean difference ranging from 2.85 to 3.10 mmHg, lower 95% confidence limit ranging from 1.05 to 1.43, upper 95% confidence limit ranging from 4.31 to 5.06), little evidence of path-specific effects through either BMI alone or both BMI and GGT, with the remaining part of the total effect attributed to a direct effect via other pathways (mean difference ranging from 5.07 to 5.25 mmHg, lower 95% confidence limit ranging from 1.35 to 1.48, upper 95% confidence limit ranging from 8.76 to 9.03). There is little variation between the eight versions of each effect. As a consequence, when we depict the 24 possible decompositions in Figure 2, they are all similar, which suggests—in this example—that conclusions about the comparative strengths of different pathways could be drawn from just one particular decomposition.

**Table 4:** Estimates, SEs, and *95*% confidence intervals for the total causal effect (TCE), followed by each of the path-specific effects we have defined. All estimates are for mean differences in SBP measured in mmHg. The results are given for three values of the sensitivity parameter 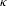: *1*, *0.5* and *0*

	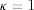		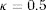		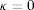	
Effect	Estimate	95% CI	Estimate	95% CI	Estimate	95% CI
TCE	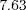	( 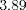 , 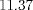 )	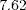	( 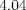 , 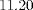 )	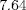	( 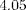 , 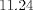 )

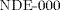	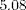	( 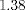 , 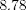 )	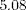	( 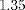 , 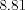 )	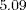	( 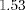 , 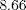 )
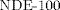	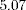	( 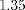 , 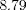 )	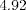	( 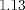 , 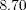 )	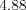	( 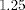 , 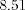 )
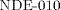	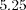	( 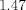 , 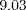 )	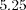	( 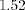 , 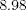 )	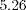	( 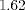 , 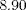 )
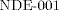	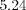	( 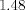 , 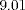 )	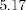	( 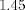 , 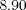 )	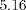	( 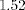 , 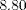 )
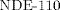	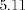	( 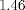 , 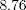 )	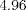	( 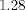 , 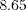 )	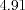	( 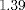 , 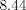 )
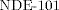	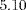	( 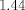 , 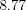 )	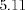	( 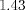 , 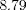 )	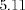	( 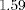 , 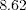 )
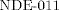	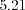	( 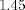 , 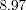 )	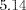	( 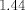 , 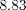 )	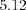	( 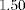 , 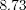 )
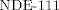	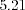	( 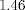 , 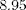 )	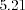	( 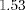 , 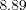 )	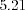	( 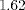 , 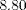 )
SNDE	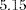	( 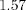 , 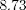 )	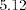	( 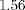 , 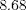 )	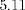	( 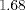 , 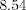 )
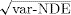	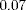		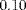		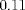	
DE^nointer^	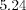	( 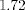 , 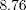 )	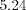	( 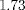 , 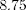 )	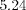	( 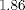 , 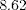 )
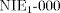	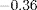	( 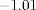 , 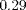 )	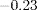	( 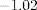 , 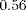 )	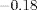	( 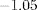 , 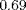 )
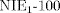	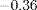	( 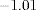 , 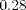 )	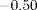	( 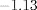 , 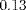 )	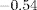	( 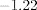 , 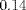 )
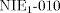	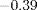	( 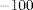 , 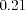 )	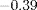	( 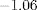 , 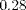 )	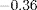	( 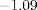 , 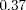 )
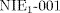	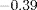	( 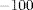 , 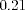 )	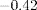	( 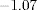 , 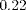 )	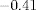	( 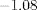 , 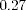 )
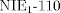	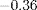	( 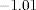 , 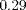 )	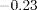	( 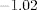 , 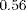 )	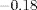	( 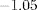 , 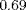 )
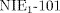	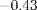	( 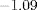 , 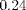 )	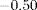	( 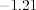 , 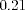 )	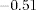	( 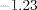 , 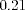 )
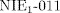	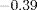	( 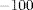 , 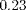 )	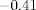	( 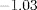 , 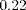 )	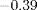	( 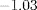 , 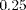 )
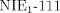	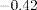	( 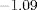 , 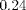 )	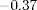	( 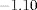 , 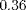 )	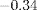	( 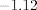 , 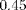 )
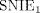	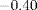	( 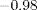 , 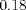 )	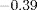	( 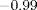 , 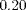 )	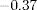	( 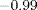 , 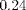 )
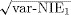	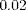		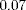		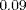	
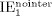	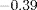	( 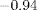 , 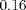 )	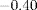	( 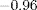 , 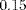 )	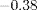	( 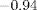 , 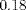 )
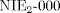	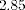	( 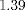 , 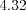 )	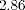	( 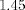 , 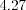 )	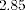	( 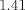 , 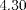 )
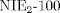	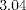	( 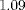 , 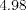 )	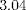	( 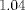 , 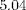 )	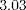	( 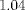 , 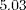 )
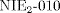	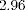	( 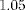 , 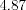 )	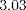	( 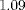 , 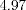 )	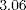	( 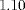 , 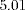 )
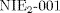	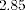	( 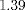 , 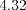 )	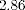	( 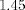 , 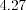 )	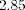	( 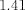 , 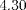 )
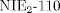	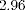	( 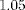 , 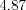 )	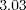	( 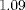 , 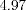 )	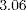	( 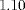 , 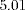 )
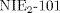	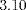	( 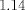 , 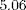 )	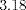	( 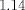 , 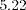 )	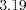	( 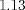 , 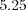 )
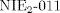	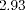	( 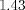 , 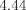 )	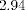	( 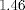 , 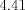 )	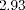	( 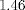 , 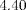 )
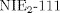	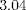	( 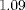 , 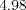 )	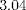	( 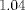 , 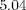 )	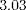	( 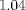 , 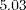 )
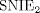	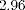	( 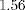 , 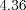 )	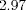	( 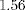 , 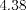 )	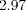	( 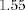 , 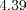 )
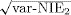	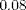		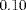		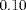	
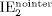	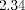	( 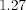 , 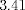 )	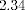	( 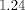 , 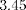 )	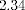	( 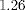 , 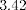 )
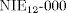	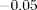	( 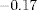 , 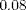 )	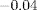	( 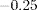 , 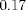 )	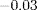	( 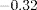 , 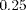 )
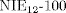	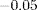	( 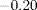 , 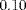 )	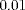	( 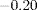 , 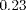 )	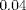	( 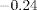 , 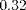 )
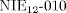	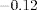	( 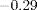 , 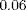 )	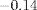	( 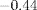 , 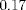 )	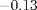	( 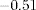 , 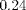 )
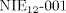	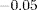	( 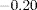 , 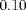 )	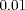	( 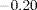 , 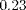 )	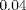	( 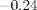 , 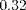 )
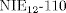	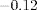	( 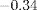 , 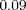 )	0000	( 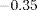 , 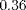 )	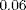	( 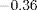 , 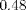 )
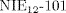	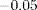	( 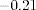 , 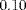 )	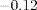	( 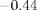 , 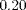 )	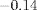	( 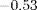 , 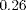 )
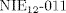	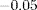	( 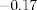 , 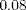 )	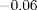	( 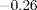 , 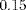 )	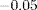	( 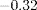 , 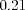 )
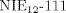	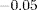	( 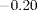 , 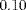 )	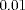	( 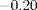 , 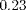 )	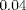	( 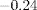 , 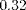 )
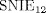	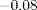	( 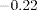 , 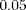 )	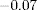	( 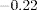 , 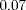 )	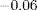	( 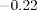 , 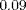 )
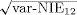	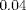		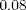		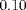	
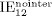	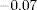	( 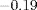 , 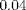 )	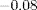	( 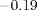 , 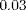 )	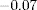	( 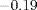 , 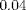 )

**Table 5:** Estimates, SEs, and *95*% confidence intervals for the total causal effect (TCE), followed by each of the mediator-specific effects we have defined. All estimates are for mean differences in SBP measured in mmHg. The results are given for three values of the sensitivity parameter 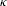: *1*, *0.5* and *0*

	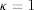		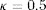		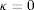	
Effect	Estimate	95% CI	Estimate	95% CI	Estimate	95% CI
TCE	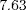	( 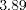 , 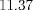 )	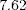	( 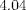 , 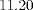 )	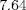	( 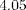 , 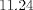 )

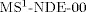	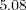	( 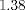 , 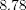 )	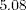	( 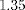 , 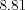 )	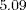	( 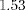 , 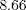 )
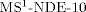	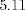	( 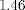 , 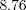 )	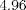	( 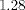 , 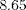 )	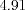	( 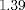 , 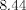 )
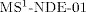	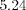	( 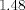 , 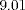 )	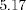	( 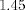 , 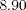 )	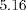	( 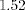 , 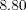 )
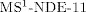	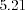	( 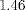 , 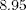 )	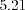	( 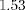 , 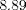 )	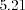	( 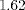 , 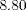 )
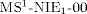	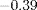	( 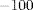 , 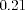 )	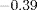	( 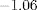 , 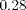 )	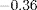	( 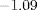 , 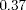 )
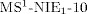	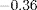	( 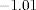 , 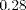 )	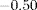	( 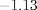 , 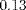 )	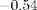	( 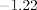 , 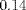 )
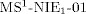	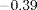	( 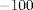 , 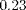 )	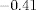	( 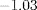 , 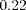 )	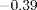	( 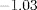 , 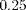 )
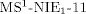	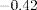	( 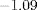 , 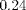 )	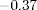	( 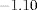 , 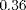 )	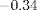	( 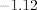 , 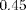 )
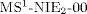	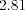	( 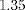 , 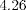 )	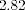	( 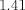 , 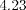 )	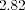	( 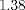 , 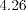 )
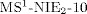	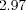	( 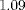 , 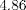 )	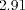	( 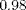 , 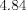 )	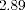	( 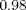 , 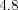 )
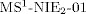	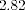	( 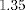 , 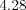 )	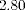	( 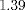 , 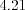 )	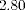	( 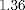 , 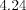 )
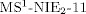	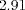	( 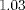 , 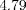 )	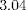	( 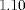 , 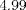 )	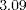	( 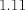 , 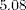 )
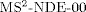	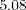	( 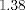 , 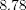 )	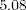	( 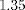 , 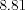 )	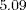	( 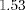 , 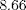 )
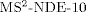	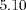	( 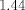 , 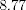 )	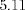	( 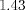 , 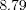 )	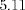	( 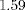 , 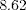 )
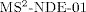	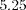	( 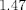 , 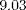 )	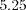	( 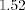 , 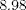 )	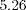	( 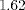 , 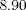 )
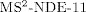	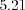	( 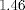 , 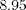 )	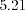	( 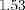 , 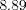 )	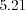	( 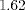 , 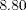 )
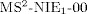	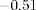	( 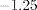 , 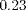 )	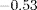	( 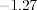 , 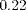 )	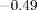	( 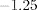 , 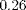 )
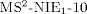	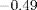	( 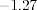 , 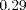 )	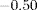	( 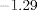 , 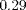 )	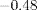	( 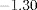 , 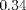 )
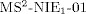	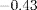	( 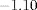 , 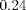 )	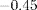	( 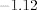 , 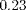 )	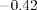	( 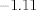 , 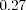 )
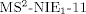	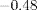	( 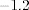 , 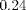 )	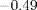	( 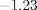 , 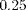 )	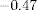	( 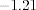 , 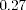 )
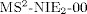	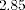	( 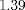 , 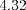 )	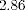	( 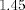 , 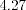 )	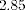	( 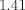 , 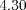 )
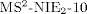	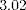	( 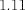 , 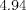 )	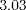	( 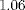 , 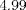 )	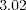	( 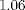 , 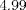 )
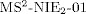	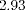	( 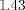 , 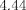 )	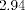	( 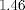 , 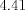 )	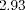	( 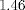 , 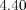 )
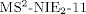	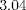	( 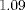 , 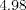 )	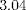	( 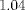 , 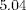 )	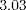	( 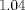 , 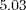 )

Due to the lack of important interactions, the summary effects included in Table 4 and Web Figure 1 are similar to each of the 8 effects in each instance. They are also similar to the results obtained when assuming no exposure–mediator interactions as implicitly done when fitting a traditional LSEM and multiplying path coefficients (note however the narrower CIs in the latter, due to the assumption of no interactions). The mediator-specific effects (Table 5 and Web Figure 2) also show a similar picture, with little difference between the two ways of defining the mediator-specific effects, due to the small magnitude of the path-specific effect through both BMI and GGT.

The results appear to be insensitive to variations in 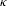 (Tables 4 and 5), and confirm that some effects do not depend on 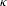 as theory suggests (see Web Figures 16–19).

### 6.4. Limitations

The exposure (heavy drinking) is likely subject to misclassification. This is of particular concern in mediation analyses, if either of the mediators (in this case GGT) is a good proxy for the true exposure, leading to an inflation of the estimated indirect effect. A feature of the Izhevsk Family Study, not exploited here, is that extremely rich information was collected (from both the subjects and a proxy) on the types, quantities and patterns of alcohol consumption. In these analyses we used only the information on estimated total ethanol consumption in one year and simplified it into a binary variable (heavy/not heavy). Concerns that the indirect effect through GGT could be inflated due to GGT's role as a good proxy for true alcohol exposure could potentially be reduced by incorporating more of the collected alcohol information.

In this setting, assumptions (T.2), (MC.2), and (MN.4) imply that age, SES and smoking are sufficient to control for confounding of the alcohol–BMI, alcohol–GGT and alcohol–SBP relationships, and that BMI and alcohol, in addition to these baseline confounders are sufficient to control for confounding of the GGT–SBP relationship. In addition, we assume that all the specified parametric models are correctly specified, and that the assumptions made regarding the missing data mechanisms justified.

## 7. Concluding Remarks

Researchers are often interested in a decomposition into multiple path-specific effects through many mediators, but due to the focus in the causal inference literature primarily on one mediator, multiple mediator analyses are typically performed using LSEM, ignoring interactive and nonlinear effects, and often ignoring the effect of one mediator on another. We have shown that extending the mediation framework to multiple mediators gives rise to complexities (in terms of multiplicity of definitions) and challenges (for identification) beyond what might have been anticipated. As well as outlining these, we have provided suggestions on how to proceed in practice, via coarser decompositions and summary effects. Important future developments include extending semiparametric estimation approaches to estimate the effects defined here.

## 8. Supplementary Materials

Web Appendices, Tables and Figures, referenced in Sections 1, 3.4–3.6, 4.2, and 6.1–6.3 are available with this paper at the *Biometrics* website on Wiley Online Library.
