# Supplementary material for: Causal Mediation Analysis with Multiple Mediators
Source: Biometrics. 2014 Oct 28;71(1):1–14. doi: 10.1111/biom.12248 (PMC4402024; doi:10.1111/biom.12248)
Supplement: Supplementary file 1 — Supplementary Materials. [file biom0071-0001-sd1.pdf]

Web-based Supplementary Materials for "Causal mediation analysis with multiple mediators"

R. M. Daniel*, **B. L. De Stavola, S. N. Cousens**

Centre for Statistical Methodology, London School of Hygiene and Tropical Medicine, Keppel Street, London, WC1E 7HT, UK.

and

S. Vansteelandt

Department of Applied Mathematics, Computer Science and Statistics, Ghent University, Belgium.

[Web Table 1 about here.]

[Web Table 2 about here.]

Key to Web Table 2:

NPI: Nonparametrically identified

set 1: Assumptions (MC.1), (MC.2), (MN.3)–(MN.5)

SP: sensitivity parameter(s)

set 1*: A variant of set 1 stated by Imai and Yamamoto (2013). In particular, the conditional independence assumptions are stated slightly differently. For more information on the implications of these subtle differences, see Hafeman and VanderWeele (2011); Robins et al. (2009); Robins and Richardson (2011).

set 2: set 1* in conjunction with a particular semiparametric additive regression model.

[Web Figure 1 about here.]

[Web Figure 2 about here.]

Web Appendix A: Causal mediation estimands with n causally-ordered mediators

First we note that there are 2^n paths from X to Y when there are n causally-ordered mediators, one for each subset of $\{M_1, \ldots, M_n\}$. Since we are interested in the finest possible decomposition, this is therefore a decomposition into 2^n path-specific effects.

We extend the notation used in the main manuscript by recursively defining $M_k^*\left(x_1, \ldots, x_{2^{k-1}}\right)$ for $k = 1, \ldots, n$ and $Y^*\left(x_1, \ldots, x_{2^n}\right)$ as follows:

$$
\begin{aligned}
M_1^*\left(x_1\right) &= M_1\left(x_1\right) \\
M_2^*\left(x_1, x_2\right) &= M_2\left(x_1, M_1^*\left(x_2\right)\right) \\
&\vdots \\
M_k^*\left(x_1, x_2, \ldots, x_{2^{k-1}}\right) &= M_k\left(x_1, M_1^*\left(x_2\right), \ldots, M_{k-1}^*\left(x_{2^{k-2}+1}, \ldots, x_{2^{k-1}}\right)\right) \\
&\vdots \\
M_n^*\left(x_1, x_2, \ldots, x_{2^{n-1}}\right) &= M_n\left(x_1, M_1^*\left(x_2\right), \ldots, M_{n-1}^*\left(x_{2^{n-2}+1}, \ldots, x_{2^{n-1}}\right)\right) \\
Y^*\left(x_1, x_2, \ldots, x_{2^n}\right) &= Y\left(x_1, M_1^*\left(x_2\right), \ldots, M_n^*\left(x_{2^{n-1}+1}, \ldots, x_{2^n}\right)\right).
\end{aligned}
$$

Then, a general causal contrast can be written as:

$$
E\left\{Y^*\left(x_1, x_2, \ldots, x_{2^n}\right) - Y^*\left(z_1, z_2, \ldots, z_{2^n}\right)\right\}.
$$

First note that this is only a path-specific effect from a finest possible decomposition if:

(1) For one value $k^* \in \{1, \ldots, 2^n\}$, $x_{k^*} = 1$ and $z_{k^*} = 0$; and

(2) For all other values of $k \in \{1, \ldots, 2^n\}$ such that $k \neq k^*$, $x_k = z_k$.

Second, note that by the way in which we constructed $Y^*(\cdot)$ above, the path through $\emptyset$ (i.e. the direct path) corresponds to $k^* = 1$, and the path through any non-empty subset $\{M_{k_1}, M_{k_2}, \ldots, M_{k_l}\}$ of $\{M_1, M_2, \ldots, M_n\}$ corresponds to

$$k^* = 1 + 2^{k_1 - 1} + 2^{k_2 - 1} + \cdots + 2^{k_l - 1}. \tag{A.1}$$

Thus we have a general definition of a path-specific effect when there are n mediators. For each path, there are $2^{(2^n-1)}$ ways of choosing how to fix the $2^n - 1$ arguments other than x_{k^*}. These correspond to one level-0 effect, $2^n - 1$ level-1 effects, $\begin{pmatrix} 2^n - 1 \\ 2 \end{pmatrix}$ level-2 effects, ..., $\begin{pmatrix} 2^n - 1 \\ k \end{pmatrix}$ level-k effects, ..., $2^n - 1$ level-$(2^n - 2)$ effects, and one level-$(2^n - 1)$ effect. There are 2^n paths, and thus $(2^n)\left\{2^{(2^n-1)}\right\} = 2^{(2^n+n-1)}$ effects in total. When $n = 2$, $2^{(2^n+n-1)} = 32$, corresponding to the number of effects given in Table 1 in the main manuscript.

Furthermore, we can construct a decomposition of the total causal effect into a sum of path-specific effects using the algorithm given below. First, we require some additional notation. Write $\mathbf{0}_a^{(b_1, b_2, \ldots, b_s)}$ for the $(1 \times a)$ row-vector with entries b_1, b_2, ..., and b_s all equal to 1, and the remaining entries equal to 0; write $\mathbf{0}_a$ for the $(1 \times a)$ zero row-vector and $\mathbf{1}_a$ for the $(1 \times a)$ row-vector consisting entirely of ones. Here is the algorithm:

(1) Choose a path, i.e. choose a subset S_1 of $\{M_1, M_2, \ldots, M_n\}$.

(2) If $S_1 = \emptyset$ then $k_1^* = 1$; otherwise, set k_1^* according to (A.1).

(3) The path-specific effect associated with S_1 is a level-0 effect and is given by:

$$\mathrm{NPSE}_{S_1} = E\left\{Y^*\left(\mathbf{0}_{2^n}^{(k_1^*)}\right) - Y^*\left(\mathbf{0}_{2^n}\right)\right\}$$

where NPSE stands for *natural path-specific effect.*

(4) Choose a second path, distinct from the first, i.e. choose a subset S_2 of $\{M_1, M_2, \ldots, M_n\}$ such that $S_2 \neq S_1$.

(5) If $S_2 = \emptyset$ then $k_2^* = 1$; otherwise, set k_2^* according to (A.1).

(6) The path-specific effect associated with S_2 is a level-1 effect and is given by:

$$\text{NPSE}_{S_2} = E\left\{Y^*\left(\mathbf{0}_{2^n}^{\left(k_1^*,k_2^*\right)}\right) - Y^*\left(\mathbf{0}_{2^n}^{\left(k_1^*\right)}\right)\right\}.$$

(7) Keep repeating these three steps. The pth path-specific effect is a level-$(p-1)$ effect through S_p and is given by

$$\text{NPSE}_{S_p} = E\left\{Y^*\left(\mathbf{0}_{2^n}^{\left(k_1^*,\ldots,k_p^*\right)}\right) - Y^*\left(\mathbf{0}_{2^n}^{\left(k_1^*,\ldots,k_{p-1}^*\right)}\right)\right\}.$$

(8) Finally, the $(2^n)^{\text{th}}$ path-specific effect is a level-(2^n-1) effect through the only remaining subset S_{2^n} and is given by

$$\text{NPSE}_{S_{2^n}} = E\left\{Y^*\left(\mathbf{0}_{2^n}^{\left(k_1^*,\ldots,k_{2^n}^*\right)}\right) - Y^*\left(\mathbf{0}_{2^n}^{\left(k_1^*,\ldots,k_{2^n-1}^*\right)}\right)\right\}$$

Note that, since $(k_1^*, \ldots, k_{2^n}^*)$ are all distinct (since each corresponds to a distinct subset/path), $(k_1^*, \ldots, k_{2^n}^*)$ contains all the integers from 1 to 2^n exactly once. Thus $\mathbf{0}_{2^n}^{\left(k_1^*,\ldots,k_{2^n}^*\right)}$ is a (1×2^n) row-vector consisting entirely of ones. That is,

$$\text{NPSE}_{S_{2^n}} = E\left\{Y^*\left(\mathbf{1}_{2^n}\right) - Y^*\left(\mathbf{0}_{2^n}^{\left(k_1^*,\ldots,k_{2^n-1}^*\right)}\right)\right\}.$$

The total causal effect decomposes into the sum of these path-specific effects:

$$\begin{aligned}
\sum_{p=1}^{2^n} \text{NPSE}_{S_p} &= E\left\{Y^*\left(\mathbf{0}_{2^n}^{\left(k_1^*\right)}\right) - Y^*\left(\mathbf{0}_{2^n}\right)\right\} \\
&\quad + E\left\{Y^*\left(\mathbf{0}_{2^n}^{\left(k_1^*,k_2^*\right)}\right) - Y^*\left(\mathbf{0}_{2^n}^{\left(k_1^*\right)}\right)\right\} \\
&\quad + \cdots \\
&\quad + E\left\{Y^*\left(\mathbf{0}_{2^n}^{\left(k_1^*,\ldots,k_{2^n-1}^*\right)}\right) - Y^*\left(\mathbf{0}_{2^n}^{\left(k_1^*,\ldots,k_{2^n-2}^*\right)}\right)\right\} \\
&\quad + E\left\{Y^*\left(\mathbf{1}_{2^n}\right) - Y^*\left(\mathbf{0}_{2^n}^{\left(k_1^*,\ldots,k_{2^n-1}^*\right)}\right)\right\} \\
&= E\left\{Y^*\left(\mathbf{1}_{2^n}\right) - Y^*\left(\mathbf{0}_{2^n}\right)\right\} = \text{TCE}. \qquad \text{(A.2)}
\end{aligned}$$

The order in which the paths were chosen $(S_1, \ldots, S_{2^n})$ was completely arbitrary, and

thus the algorithm above can be used to give rise to $(2^n)!$ decompositions, one for each permutation of $(S_1,\ldots,S_{2^n})$. Each decomposition consists of exactly one level-0, one level-1, ..., one level-(2^n-2), and one level-(2^n-1) effect—and these types are allocated to the paths in this order. A non-trivial permutation of $(S_1,\ldots,S_{2^n})$ thus leads to at least two of the path-specific effects changing their type, and thus each of the $(2^n)!$ decompositions constructed via the algorithm above is distinct. Furthermore, there can be no decompositions other than the $(2^n)!$ constructed in this way. This is because the necessary cancelling out of terms that happens in (A.2) requires that each $Y^*\left(\mathbf{0}_{2^n}^{(k_1^*,\ldots,k_p^*)}\right)$ that appears as the left-hand term of one effect, must also appear as the right-hand term of another effect; and for this second effect to be a path-specific effect, the right-hand term in question can differ from its corresponding left-hand term only in one entry. Thus, the choice of the order in which the paths appear in the algorithm is the *only* free choice one has in constructing effects that permit decomposition.

Web Appendix B: Example: Linear models with interactions

From the linear model with interactions (assuming no confounders) given in Section 3.5 of the main manuscript, we derive the following potential mediators and outcomes:

$$M_1(x)=\alpha_0+\alpha_x x+\varepsilon_{m_1}^{x}$$

where $\varepsilon_{m_1}^{x}=M_1(x)-E\left\{M_1(x)\right\}$,

$$M_2(x,M_1(x'))=\beta_0+\beta_x x+(\beta_{m_1}+\beta_{xm_1}x)\left(\alpha_0+\alpha_x x'+\varepsilon_{m_1}^{x'}\right)+\varepsilon_{m_2}^{xx'}$$

where $\varepsilon_{m_2}^{xx'}=M_2(x,M_1(x'))-E\left\{M_2(x,M_1(x'))\right\}$, and

$$\begin{aligned}Y(x,M_1(x'),M_2(x'',M_1(x'''))) &= \gamma_0+\gamma_x x+(\gamma_{m_1}+\gamma_{xm_1}x)\left(\alpha_0+\alpha_x x'+\varepsilon_{m_1}^{x'}\right)\\ &\quad+\left\{\gamma_{m_2}+\gamma_{xm_2}x+(\gamma_{m_1m_2}+\gamma_{xm_1m_2}x)\left(\alpha_0+\alpha_x x'+\varepsilon_{m_1}^{x'}\right)\right\}\\ &\quad\cdot\left\{\beta_0+\beta_x x''+(\beta_{m_1}+\beta_{xm_1}x'')\left(\alpha_0+\alpha_x x'''+\varepsilon_{m_1}^{x'''}\right)+\varepsilon_{m_2}^{x''x'''}\right\}+\varepsilon_{y}^{xx'x''x'''}\end{aligned}$$

where $\varepsilon_y^{xx'x''x'''} = Y(x, M_1(x'), M_2(x'', M_1(x'''))) - E\{Y(x, M_1(x'), M_2(x'', M_1(x''')))\}$.

This leads to the following expressions for the 32 effects in Table 1 in the main manuscript:

$$\text{NDE-}x'x''x''' = \gamma_x + \gamma_{xm_1}(\alpha_0 + \alpha_x x')$$
$$+ \{\gamma_{xm_2} + \gamma_{xm_1m_2}(\alpha_0 + \alpha_x x')\}\{\beta_0 + \beta_x x'' + (\beta_{m_1} + \beta_{xm_1}x'')(\alpha_0 + \alpha_x x''')\}$$
$$+ \gamma_{xm_1m_2}(\beta_{m_1} + \beta_{xm_1}x'')E\left(\varepsilon_{m_1}^{x'}\varepsilon_{m_1}^{x'''}\right),$$

$$\text{NIE}_1\text{-}xx''x''' = \alpha_x(\gamma_{m_1} + \gamma_{xm_1}x)$$
$$+ \alpha_x(\gamma_{m_1m_2} + \gamma_{xm_1m_2}x)\{\beta_0 + \beta_x x'' + (\beta_{m_1} + \beta_{xm_1}x'')(\alpha_0 + \alpha_x x''')\}$$
$$+ (\gamma_{m_1m_2} + \gamma_{xm_1m_2}x)(\beta_{m_1} + \beta_{xm_1}x'')E\left\{\left(\varepsilon_{m_1}^1 - \varepsilon_{m_1}^0\right)\varepsilon_{m_1}^{x'''}\right\},$$

$$\text{NIE}_2\text{-}xx'x''' = \{\gamma_{m_2} + \gamma_{xm_2}x + (\gamma_{m_1m_2} + \gamma_{xm_1m_2}x)(\alpha_0 + \alpha_x x')\}\cdot$$
$$\{\beta_x + \beta_{xm_1}(\alpha_0 + \alpha_x x''')\}$$
$$+ \beta_{xm_1}(\gamma_{m_1m_2} + \gamma_{xm_1m_2}x)E\left(\varepsilon_{m_1}^{x'}\varepsilon_{m_1}^{x'''}\right)$$

and

$$\text{NIE}_{12}\text{-}xx'x'' = \alpha_x(\beta_{m_1} + \beta_{xm_1}x'')\cdot$$
$$\{\gamma_{m_2} + \gamma_{xm_2}x + (\gamma_{m_1m_2} + \gamma_{xm_1m_2}x)(\alpha_0 + \alpha_x x')\}$$
$$+ (\gamma_{m_1m_2} + \gamma_{xm_1m_2}x)(\beta_{m_1} + \beta_{xm_1}x'')E\left\{\varepsilon_{m_1}^{x'}\left(\varepsilon_{m_1}^1 - \varepsilon_{m_1}^0\right)\right\}.$$

Note that the variance–covariance terms $E\left(\varepsilon_{m_1}^{x'}\varepsilon_{m_1}^{x'''}\right)$ turn out to play a key role in identification (see Section 4.2.2 of the main manuscript, and Web Appendix J).

Web Appendix C: On the interpretation of summary path-specific effects with a single mediator

In Section 3.6.2 of the main manuscript, we introduce summary path-specific effects for the two mediator setting. These expressions turn out to be much simpler for the single mediator

setting Kuha and Golthorpe (2010). They are:

$$\text{SNDE} = \frac{1}{2}\text{PNDE} + \frac{1}{2}\text{TNDE}$$
$$\text{SNIE} = \frac{1}{2}\text{PNIE} + \frac{1}{2}\text{TNIE}$$

also giving

$$\text{SNDE} + \text{SNIE} = \text{TCE}$$

In a randomised experiment, with $Pr(X = 1) = \frac{1}{2}$, the SNDE, i.e. the average of the PNDE and TNDE, can be interpreted as the effect that would have been observed if everyone had stayed at his or her observed mediator level, regardless of treatment arm, i.e. $E\{Y(1,M) - Y(0,M)\}$. This is because $Y(1,M) - Y(0,M)$ is equal to $Y(1,M(0)) - Y(0,M(0))$ in the $X = 0$ group, and $Y(1,M(1)) - Y(0,M(1))$ in the $X = 1$ group, and thus the average of $Y(1,M) - Y(0,M)$ is equal to the average of the PNDE and TNDE.

In a non-randomised study, the SNDE could still be interpreted as the effect that would be found in such hypothetical randomised experiment.

Web Appendix D: Comparison of summary natural and LSEM-based path-specific effects

To show that interactions can influence the values of the summary natural path-specific effects, we consider the following very simple example. Suppose that the data generating process is $X \sim N(0,1)$, $M|X \sim N(X,1)$ and $Y|X,M \sim N(XM,1)$. This leads to $E(Y(0,M(0))) = E(Y(0,M(1))) = E(Y(1,M(0))) = 0$ and $E(Y(1,M(1))) = 1$. This implies that PNDE=PNIE=0 and TNDE=TNIE=TCE=1. This gives summary natural direct and indirect effects of SNDE=SNIE=0.5. However, if we wrongly assume there to be no XM interaction in the model for Y our assumed (incorrect) model would be:

$$E(Y|X,M) = \gamma_0 + \gamma_x X + \gamma_m M$$

The values of $(\gamma_0, \gamma_x, \gamma_m)$ satisfy:

$$0 = E\left\{\begin{pmatrix} 1 \\ X \\ M \end{pmatrix}(Y - \gamma_0 - \gamma_x X - \gamma_m M)\right\}$$

$$= E\left[E\left\{\begin{pmatrix} 1 \\ X \\ M \end{pmatrix}(Y - \gamma_0 - \gamma_x X - \gamma_m M)\middle| X, M\right\}\right]$$

$$= E\left\{\begin{pmatrix} 1 \\ X \\ M \end{pmatrix}(XM - \gamma_0 - \gamma_x X - \gamma_m M)\right\}$$

$$= E\left[E\left\{\begin{pmatrix} 1 \\ X \\ M \end{pmatrix}(XM - \gamma_0 - \gamma_x X - \gamma_m M)\middle| X\right\}\right]$$

$$= E\left[E\left\{\begin{pmatrix} XM - \gamma_0 - \gamma_x X - \gamma_m M \\ X^2 M - \gamma_0 X - \gamma_x X^2 - \gamma_m XM \\ XM^2 - \gamma_0 M - \gamma_x XM - \gamma_m M^2 \end{pmatrix}\middle| X\right\}\right]$$

$$= E\left\{\begin{pmatrix} X^2 - \gamma_0 - \gamma_x X - \gamma_m X \\ X^3 - \gamma_0 X - \gamma_x X^2 - \gamma_m X^2 \\ X\left(1 + X^2\right) - \gamma_0 X - \gamma_x X^2 - \gamma_m \left(1 + X^2\right) \end{pmatrix}\right\}$$

(since $E\left(M|X\right) = X$ and $E\left(M^2|X\right) = 1 + X^2$)

$$= \begin{pmatrix} 1 - \gamma_0 \\ -\gamma_x - \gamma_m \\ -\gamma_x - 2\gamma_m \end{pmatrix}$$

(since $E\left(X\right) = E\left(X^3\right) = 0$ and $E\left(X^2\right) = 1$)

This implies that $\gamma_0 = 1$ and $\gamma_x = \gamma_m = 0$. Thus, if we wrongly assume there to be no XM interaction in the model for Y, we would obtain direct, indirect and total effects all equal to 0.

Web Appendix E: Identification of the CDE with two mediators

In Section 4.2.1 of the main manuscript, we mention that the CDE with two mediators can be identified, under assumptions (MC.1) and (MC.2) using the g-computation formula (Robins, 1986). The identification formula is given below:

$$
\begin{aligned}
&E\left\{Y\left(1, m_1, m_2\right)-Y\left(0, m_1, m_2\right)\right\}= \\
&\qquad \int_{\mathcal{C}} \int_{\mathcal{L}_1} \int_{\mathcal{L}_2} \left\{E\left(Y \left| C=c, X=1, L_1=l_1, M_1=m_1, L_2=l_2, M_2=m_2\right.\right) \cdot\right. \\
&\qquad\qquad f_{L_1|C,X}\left(l_1 \left| c, 1\right.\right) f_{L_2|C,X,L_1,M_1}\left(l_2 \left| c, 1, l_1, m_1\right.\right) \\
&\qquad\qquad -E\left(Y \left| C=c, X=0, L_1=l_1, M_1=m_1, L_2=l_2, M_2=m_2\right.\right) \cdot \\
&\qquad\qquad \left. f_{L_1|C,X}\left(l_1 \left| c, 0\right.\right) f_{L_2|C,X,L_1,M_1}\left(l_2 \left| c, 0, l_1, m_1\right.\right)\right\} \cdot \\
&\qquad\qquad f_C(c)\, d\mu_{L_2}\left(l_2\right) d\mu_{L_1}\left(l_1\right) d\mu_C(c) . \qquad \text{(A.3)}
\end{aligned}
$$

Web Appendix F: On assumption (MN.5)

Consider the setting in which the data generating process is a NPSEM:

$$X = g_X\left(C, U_X\right) \qquad \text{(A.4)}$$

$$L_1 = g_{L_1}\left(C, X, U_{L_1}\right) \qquad \text{(A.5)}$$

$$M_1 = g_{M_1}\left(C, X, L_1, U_{M_1}\right) \qquad \text{(A.6)}$$

$$L_2 = g_{L_2}\left(C, X, L_1, M_1, U_{L_2}\right) \qquad \text{(A.7)}$$

$$M_2 = g_{M_2}\left(C, X, L_1, M_1, L_2, U_{M_2}\right) \qquad \text{(A.8)}$$

$$Y = g_Y\left(C, X, L_1, M_1, L_2, M_2, U_Y\right) \qquad \text{(A.9)}$$

where $g_{\cdot}(\cdot)$ are deterministic functions and $\{C, U_X, U_{L_1}, U_{M_1}, U_{L_2}, U_{M_2}, U_Y\}$ are mutually independent. Consider the following counterfactuals derived from the model above:

$$M_1(x) = g_{M_1}(C, x, g_{L_1}(C, x, U_{L_1}), U_{M_1}) \tag{A.10}$$

$$M_1(x^*) = g_{M_1}(C, x^*, g_{L_1}(C, x^*, U_{L_1}), U_{M_1}) \tag{A.11}$$

$$M_2(x, m_1) = g_{M_2}(C, x, g_{L_1}(C, x, U_{L_1}), m_1, g_{L_2}(C, x, g_{L_1}(C, x, U_{L_1}), m_1, U_{L_2}), U_{M_2}) \tag{A.12}$$

$$M_2(x^*, m_1^*) = \\ g_{M_2}(C, x^*, g_{L_1}(C, x^*, U_{L_1}), m_1^*, g_{L_2}(C, x^*, g_{L_1}(C, x^*, U_{L_1}), m_1^*, U_{L_2}), U_{M_2}) \tag{A.13}$$

$$Y(x, m_1, m_2) = g_Y(C, x, g_{L_1}(C, x, U_{L_1}), m_1, g_{L_2}(C, x, g_{L_1}(C, x, U_{L_1}), m_1, U_{L_2}), m_2, U_Y) \tag{A.14}$$

And consider the following conditional independence statements, for $x = 0, 1$, $x^* = 0, 1$, $x' = 0, 1$ and $\forall c, l_1, l_2, m_1, m_2, m_1'$:

$$M_2(x, m_1) \perp\!\!\!\perp \{M_1(x), M_1(x^*)\} | C = c, L_1 = l_1 , \tag{A.15}$$

$$Y(x, m_1, m_2) \perp\!\!\!\perp \{M_1(x), M_1(x^*)\} | C = c, L_1 = l_1 \tag{A.16}$$

and

$$Y(x, m_1, m_2) \perp\!\!\!\perp M_2(x', m_1') | C = c, L_1 = l_1, L_2 = l_2, M_1(x) = m_1, M_1(x^*) = m_1' . \tag{A.17}$$

Conditional independence (A.15) is violated by the NPSEM above since (A.10)–(A.12) all involve U_{L_1}. Similarly, conditional independence (A.16) is violated since (A.10), (A.11) and (A.14) all involve U_{L_1}. Finally, conditional independence (A.17) is violated since (A.12)–(A.14) all involve both U_{L_1} and U_{L_2}.

On the other hand, if we omit intermediate confounders L_1 and L_2, the NPSEM becomes $X = g_X(C, U_X)$, $M_1 = g_{M_1}(C, X, U_{M_1})$, $M_2 = g_{M_2}(C, X, M_1, U_{M_2})$ and $Y =$

$g_Y(C, X, M_1, M_2, U_Y)$, giving the following counterfactuals:

$$M_1(x) = g_{M_1}(C, x, U_{M_1}) \quad \text{(A.18)}$$

$$M_1(x^*) = g_{M_1}(C, x^*, U_{M_1}) \quad \text{(A.19)}$$

$$M_2(x, m_1) = g_{M_2}(C, x, m_1, U_{M_2}) \quad \text{(A.20)}$$

$$M_2(x^*, m_1^*) = g_{M_2}(C, x^*, m_1^*, U_{M_2}) \quad \text{(A.21)}$$

$$Y(x, m_1, m_2) = g_Y(C, x, m_1, m_2, U_Y) \quad \text{(A.22)}$$

After conditioning on C, we see that {(A.18), (A.19)} are independent of (A.20), and of (A.22), and that {(A.20), (A.21)} are independent of (A.22); that is, assumption (MN.5) implies that A.15, A.16 and A.17 hold. It is this fact that aids identification (see the proof of Theorem 1 given in Web Appendix H).

See Figure 4 in Web Appendix G for an example of part of this algebraic argument displayed graphically.

Web Appendix G: Cross-world directed acyclic graphs

In this section, we provide two examples of cross-world directed acyclic graphs (Shpitser and Pearl, 2008) which complement the arguments given, particularly with respect to intermediate confounding, in Section 4 of the main manuscript and the relevant Web Appendices referenced therein. Worlds in which different interventions are applied are drawn separately, but correlations between variables across different worlds are explicitly shown using the "U" variables. d-separation (Pearl, 1995) can then be applied to read off from the graph which conditional independence relationships between counterfactual variables hold. Similar graphs corresponding to all the other algebraic arguments are omitted in the interest of brevity, but can be obtained from the corresponding author on request.

[Web Figure 3 about here.]

[Web Figure 4 about here.]

Web Appendix H: Proof of Theorem 1

$$
\begin{aligned}
&E\left\{Y\left(x, M_1(x'), M_2(x'', M_1(x'''))\right)\right\} \\
&\quad = \int_{\mathcal{C}} E\left\{Y\left(x, M_1(x'), M_2(x'', M_1(x'''))\right) \left| C = c\right.\right\} f_C\left(c\right) d\mu_C\left(c\right) \\
&\quad = \int_{\mathcal{C}} \int_{\mathcal{M}_1} \int_{\mathcal{M}_1} \int_{\mathcal{M}_2} E\left\{Y\left(x, m_1, m_2\right) \left| C = c, M_1(x') = m_1, M_2(x'', m_1') = m_2,\right.\right. \\
&\qquad\quad \left. M_1(x''') = m_1'\right\} f_{M_2(x'', m_1')|C, M_1(x'''), M_1(x')}\left(m_2 \left| c, m_1', m_1\right.\right) \\
&\qquad\qquad\qquad \cdot f_{M_1(x''')|C, M_1(x')}\left(m_1' \left| c, m_1\right.\right) f_{M_1(x')|C}\left(m_1 \left| c\right.\right) f_C\left(c\right) \\
&\qquad\qquad\qquad\qquad \cdot d\mu_{M_2}\left(m_2\right) d\mu_{M_1}\left(m_1'\right) d\mu_{M_1}\left(m_1\right) d\mu_C\left(c\right)
\end{aligned}
$$

$$
\begin{aligned}
&\quad = \int_{\mathcal{C}} \int_{\mathcal{M}_1} \int_{\mathcal{M}_1} \int_{\mathcal{M}_2} E\left\{Y\left(x, m_1, m_2\right) \left| C = c\right.\right\} f_{M_2(x'', m_1')|C}\left(m_2 \left| c\right.\right) \\
&\qquad\qquad \cdot f_{M_1(x''')|C, M_1(x')}\left(m_1' \left| c, m_1\right.\right) f_{M_1(x')|C}\left(m_1 \left| c\right.\right) f_C\left(c\right) \\
&\qquad\qquad\qquad \cdot d\mu_{M_2}\left(m_2\right) d\mu_{M_1}\left(m_1'\right) d\mu_{M_1}\left(m_1\right) d\mu_C\left(c\right) \qquad \text{(A.23)} \\
&\quad = \int_{\mathcal{C}} \int_{\mathcal{M}_1} \int_{\mathcal{M}_1} \int_{\mathcal{M}_2} E\left\{Y \left| C = c, X = x, M_1 = m_1, M_2 = m_2\right.\right\} \\
&\qquad \cdot f_{M_2|C,X,M_1}\left(m_2 \left| c, x'', m_1'\right.\right) \boxed{f_{M_1(x''')|C, M_1(x')}\left(m_1' \left| c, m_1\right.\right)} f_{M_1|C,X}\left(m_1 \left| c, x'\right.\right) \\
&\qquad\qquad\qquad \cdot f_C\left(c\right) d\mu_{M_2}\left(m_2\right) d\mu_{M_1}\left(m_1'\right) d\mu_{M_1}\left(m_1\right) d\mu_C\left(c\right) \qquad \text{(A.24)}
\end{aligned}
$$

where (A.23) follows from assumption (MN.5) (together with the conditional independence implications given in Web Appendix F) and (A.24) follows from assumptions (MC.2) and (MN.4).

Web Appendix J: Identification and sensitivity analysis under a particular parametric model

When there is an effect of M_1 on M_2, we saw in Section 4.2.1 of the main manuscript that eight of the 32 effects listed in Table 1 in the main manuscript, together with two of the first set of mediator-specific effects, and all of the second set of mediator-specific effects are nonparametrically identified under assumptions (MC.1), (MC.2) and (MN.3)–(MN.5). The remaining effects require knowledge of the boxed density in equation (4) of the main manuscript when $x' \neq x'''$. Under the assumption of strong stability, and under particular specifications of the SEM for this system, this joint distribution is identified. For example, suppose the structural equation for M_1 is of the form:

$$M_1 = g_{M_1}(C, X, U_{M_1}) \equiv h(C, X) + U_{M_1} \tag{A.25}$$

for some function $h(C, X)$, then

$$f_{M_1(x''')|C,M_1(x')}(m_1' | c, m_1) = I\{m_1' = m_1 + h(c, x''') - h(c, x')\}.$$

On the other hand, if the structural equation for M_1 is of the form:

$$M_1 = g_{M_1}(C, X, U_{M_1}) \equiv h(C, X) + U_{M_1,0}(1 - X) + U_{M_1,1}X, \tag{A.26}$$

where $U_{M_1} = (U_{M_1,0}, U_{M_1,1})$ and $U_{M_1,0}$ and $U_{M_1,1}$ are independent then, for $x' \neq x'''$,

$$f_{M_1(x''')|C,M_1(x')}(m_1' | c, m_1) = f_{M_1(x''')|C}(m_1' | c) = f_{M_1|C,X}(m_1' | c, x'''),$$

with the second equality justified under assumption (MN.4).

The (semiparametric) SEMs in (A.25) and (A.26) represent the situations in which $M_1(0)$ and $M_1(1)$ are perfectly correlated and independent (given C), respectively. We may not believe either of these extremes to be appropriate. By specifying the form of the SEM more restrictively, we can also find SEMs for which the conditional correlation of $M_1(0)$ and $M_1(1)$ given C is between 0 and 1, and for which the boxed quantity in equation (4) of the main manuscript is identified up to a sensitivity parameter. For example, consider the following

form for the SEM for M_1:

$$M_1 = g_{M_1}(C, X, U_{M_1}) \equiv h(C, X) + U_{M_1,0}(1 - X) + U_{M_1,1}X + U_{M_1,2},$$

where $U_{M_1} = (U_{M_1,0}, U_{M_1,1}, U_{M_1,2})$ and

$$\begin{pmatrix} U_{M_1,0} \\ U_{M_1,1} \\ U_{M_1,2} \end{pmatrix} \sim N\left(\begin{pmatrix} 0 \\ 0 \\ 0 \end{pmatrix}, \sigma^2 \begin{pmatrix} 1-\kappa^2 & 0 & 0 \\ 0 & 1-\kappa^2 & 0 \\ 0 & 0 & \kappa^2 \end{pmatrix}\right).$$

Then

$$M_1(1)\,|M_1(0), C \sim N\left(h(c,1) + \kappa^2 (M_1(0) - h(c,0)), \left(1-\kappa^4\right)\sigma^2\right).$$

Note that

$$\sigma^2 = \mathrm{Var}\left(M_1\,|C, X\right)$$

can be estimated from the data. However, the data contain no information on κ^2, the proportion of residual variance shared across worlds; this becomes the sensitivity parameter, to be varied from 0 to 1. An example of this sort of sensitivity analysis is given in Section 6 of the main manuscript.

If M_1 were binary, a similar approach could be taken, where the marginal probabilities

$$Pr\left(M_1(x) = 1\,|C\right)$$

could be identified using

$$Pr\left(M_1 = 1\,|C, X = x\right)$$

and a sensitivity parameter could then be defined for

$$Pr\left(M_1(x) = 1\,|M_1(1-x) = 1, C\right),$$

respecting the fact that this probability lies within

$$\left[0, \min\left(1, \frac{Pr\left(M_1(x) = 1\,|C\right)}{Pr\left(M_1(1-x) = 1\,|C\right)}\right)\right].$$

A similar approach was taken for discrete mediators by Albert and Nelson (2011) and in a different context by Roy et al. (2008).

Web Appendix K: Identification for the linear model with interactions

Looking back at the expressions for the path-specific effects given in Web Appendix B, we see that the identification of the effects for the linear model with interactions given in Section 3.5 of the main manuscript simplifies if some of these interactions are absent, and also in the absence of an effect of M_1 on M_2 (in accordance with the more general result in Section 4.2.1 of the main manuscript).

For the NDE effects, we see that the sensitivity analysis would not be required if $\gamma_{xm_1m_2} = 0$, i.e. in the absence of a three-way interaction in the model for Y. Also, if $\beta_{m_1} = \beta_{xm_1} = 0$, corresponding to no effect of M_1 on M_2, then, as we would expect, the sensitivity analysis is not needed.

For the NIE_1 and NIE_{12} effects, we see that the sensitivity analysis would not be required if $\gamma_{m_1m_2} = \gamma_{xm_1m_2} = 0$, i.e. in the absence of both the two-way interaction between M_1 and M_2 and the three-way interaction in the model for Y. Again, if $\beta_{m_1} = \beta_{xm_1} = 0$, corresponding to no effect of M_1 on M_2, then, as we would expect, the sensitivity analysis is not needed.

Finally, for the NIE_2 effects, the sensitivity analysis would again not be required if $\gamma_{m_1m_2} = \gamma_{xm_1m_2} = 0$. And if $\beta_{xm_1} = 0$, corresponding to no interaction between X and M_1 in their effect on M_2, (even if there is a main effect of M_1 on M_2), then the sensitivity analysis is not needed.

Web Appendix L: Identification when there is intermediate confounding

Thus far, we have assumed that there be no intermediate confounding, i.e. no confounders L_1 of the M_1–M_2 or M_1–Y relationships that are affected by X, and no confounders L_2 of the M_2–Y relationship affected by either X, M_1 or both. In the case of a single mediator, intermediate confounders L of M–Y affected by X can be accommodated under a particular parametric assumption suggested by Petersen et al. (2006). We now show that generalisations

of this assumption can be used to accommodate a certain pattern of intermediate confounding when there are two causally-ordered mediators.

We consider replacing assumption (MN.5) by:

Assumption (MN.5b).

For $x = 0, 1$, $x' = 0, 1$, $x'' = 0, 1$ and $\forall c, m_1, m_2, m_1', m_2', m_1''$:

$$M_2(x, m_1) \perp\!\!\!\perp \{M_1(x), M_1(x')\} | C = c \,, \tag{A.27}$$

$$M_2(x, m_1) \perp\!\!\!\perp \{M_1(x), M_1(x')\} | C = c, M_2(x', m_1') = m_2 \,, \tag{A.28}$$

$$\begin{aligned} E\{Y(1, m_1, m_2) - Y(0, m_1, m_2) | C = c, M_1(x) = m_1, M_1(x'') = m_1', M_2(x', m_1') = m_2\} \\ = E\{Y(1, m_1, m_2) - Y(0, m_1, m_2) | C = c\} \,, \end{aligned}$$

$$\begin{aligned} E\{Y(x, m_1, m_2) - Y(x, m_1', m_2) | C = c, M_1(1) = m_1, M_1(0) = m_1', M_2(x', m_1'') = m_2\} \\ = E\{Y(x, m_1, m_2) - Y(x, m_1', m_2) | C = c\} \,, \end{aligned}$$

and

$$\begin{aligned} E\{Y(x, m_1, m_2) - Y(x, m_1, m_2') | C = c, M_1(x') = m_1, M_1(x'') = m_1', M_2(1, m_1') = m_2, \\ M_2(0, m_1') = m_2'\} = E\{Y(x, m_1, m_2) - Y(x, m_1, m_2') | C = c\} \,. \end{aligned}$$

□

Conditional independence statements (A.27) and (A.28) place a restriction on the permitted pattern of intermediate confounding. To see why, we again consider the NPSEM (A.4)–(A.9). All of (A.10)–(A.12) contain U_{L_1}, and thus (A.27) and (A.28) are violated by this NPSEM. However, if we consider instead the NPSEM where there is no effect of L_1 on

L_2 or on M_2 (Figure 5 B):

$$X = g_X\left(C, U_X\right) \tag{A.29}$$

$$L_1 = g_{L_1}\left(C, X, U_{L_1}\right) \tag{A.30}$$

$$M_1 = g_{M_1}\left(C, X, L_1, U_{M_1}\right) \tag{A.31}$$

$$L_2 = g_{L_2}\left(C, X, M_1, U_{L_2}\right) \tag{A.32}$$

$$M_2 = g_{M_2}\left(C, X, M_1, L_2, U_{M_2}\right) \tag{A.33}$$

$$Y = g_Y\left(C, X, L_1, M_1, L_2, M_2, U_Y\right) \tag{A.34}$$

which gives rise to the following counterfactuals:

$$\begin{aligned}
M_1(x) &= g_{M_1}\left(C, x, g_{L_1}\left(C, x, U_{L_1}\right), U_{M_1}\right) \\
M_1(x^*) &= g_{M_1}\left(C, x^*, g_{L_1}\left(C, x^*, U_{L_1}\right), U_{M_1}\right) \\
M_2(x, m_1) &= g_{M_2}\left(C, x, m_1, g_{L_2}\left(C, x, m_1, U_{L_2}\right), U_{M_2}\right) \\
M_2(x^*, m_1^*) &= g_{M_2}\left(C, x^*, m_1^*, g_{L_2}\left(C, x^*, m_1^*, U_{L_2}\right), U_{M_2}\right) \\
Y(x, m_1, m_2) &= g_Y\left(C, x, g_{L_1}\left(C, x, U_{L_1}\right), m_1, g_{L_2}\left(C, x, m_1, U_{L_2}\right), m_2, U_Y\right).
\end{aligned} \tag{A.35}$$

then both (A.27) and (A.28) are now satisfied, since (A.35) is no longer a function of U_{L_1}.

[Web Figure 5 about here.]

It remains to show that, under assumptions (T.2), (MC.2), (MN.4) and (MN.5b), permitting intermediate confounders as shown in Figure 5 B, all the effects defined in Section 3 of the main manuscript are identified up to some sensitivity parameters, similar to those discussed in Web Appendix J. We do this separately for (a) direct effects, (b) indirect effects through M_1 alone, and (c) indirect effects through M_2 alone. The indirect effects through both M_1 and M_2 are then identified from these effects, and the total effect (identified under assumption (T.2) as usual), by subtraction using the decompositions given in Table 2 in the main manuscript.

(a) identifying natural direct effects.

Each NDE (see Table 1 in the main manuscript) is of the form $E\left\{Y\left(1, M_1(x), M_2(x', M_1(x''))\right) - Y\left(0, M_1(x), M_2(x', M_1(x''))\right)\right\}$ for some x, x', x''. This can be re-written as:

$$
\begin{aligned}
&E\left\{Y\left(1, M_1(x), M_2(x', M_1(x''))\right) - Y\left(0, M_1(x), M_2(x', M_1(x''))\right)\right\} \\
&\quad = \int_{\mathcal{C}}\int_{\mathcal{M}_1}\int_{\mathcal{M}_1}\int_{\mathcal{M}_2} E\left\{Y\left(1, M_1(x), M_2(x', M_1(x''))\right)\right. \\
&\quad - Y\left(0, M_1(x), M_2(x', M_1(x''))\right) \left| C = c, M_1(x) = m_1, M_2(x', m_1') = m_2,\right. \\
&\qquad\quad \left. M_1(x'') = m_1'\right\} f_{M_2(x', m_1')|C, M_1(x''), M_1(x)}\left(m_2 \left| c, m_1', m_1\right.\right) \\
&\qquad\qquad\qquad\qquad \cdot f_{M_1(x'')|C, M_1(x)}\left(m_1' \left| c, m_1\right.\right) f_{M_1(x)|C}\left(m_1 \left| c\right.\right) f_C\left(c\right) \\
&\qquad\qquad\qquad\qquad\qquad\qquad \cdot d\mu_{M_2}\left(m_2\right) d\mu_{M_1}\left(m_1'\right) d\mu_{M_1}\left(m_1\right) d\mu_C\left(c\right) \\
&\quad = \int_{\mathcal{C}}\int_{\mathcal{M}_1}\int_{\mathcal{M}_1}\int_{\mathcal{M}_2} E\left\{Y\left(1, m_1, m_2\right) - Y\left(0, m_1, m_2\right) \left| C = c\right.\right\} f_{M_2(x', m_1')|C}\left(m_2 \left| c\right.\right) \\
&\qquad\qquad \cdot f_{M_1(x'')|C, M_1(x)}\left(m_1' \left| c, m_1\right.\right) f_{M_1(x)|C}\left(m_1 \left| c\right.\right) f_C\left(c\right) \\
&\qquad\qquad\qquad\qquad \cdot d\mu_{M_2}\left(m_2\right) d\mu_{M_1}\left(m_1'\right) d\mu_{M_1}\left(m_1\right) d\mu_C\left(c\right) \qquad\qquad \text{(A.36)} \\
&\quad = \int_{\mathcal{C}}\int_{\mathcal{M}_1}\int_{\mathcal{M}_1}\int_{\mathcal{M}_2} E\left\{Y\left(1, m_1, m_2\right) - Y\left(0, m_1, m_2\right) \left| C = c\right.\right\} \\
&\qquad \cdot f_{M_2|C,X,M_1}\left(m_2 \left| c, x', m_1'\right.\right) \boxed{f_{M_1(x'')|C, M_1(x)}\left(m_1' \left| c, m_1\right.\right)} f_{M_1|C,X}\left(m_1 \left| c, x\right.\right) \\
&\qquad\qquad\qquad\qquad \cdot f_C\left(c\right) d\mu_{M_2}\left(m_2\right) d\mu_{M_1}\left(m_1'\right) d\mu_{M_1}\left(m_1\right) d\mu_C\left(c\right). \qquad\qquad \text{(A.37)}
\end{aligned}
$$

(A.36) follows from assumption (MN.5b). That $f_{M_1(x)|C}\left(m_1 \left| c\right.\right)$ can be re-written as $f_{M_1|C,X}\left(m_1 \left| c, x\right.\right)$ in (A.37) follows from assumption (MN.4); whereas re-writing $f_{M_2(x', m_1')|C}\left(m_2 \left| c\right.\right)$ as $f_{M_2|C,X,M_1}\left(m_2 \left| c, x', m_1'\right.\right)$ follows from assumptions (MN.4) and (MN.5b) together. Specifically, (A.27) together with the identity $M_1 \equiv XM_1(1) + (1 - X)\, M_1(0)$ imply that, conditional on X, $M_2(x, m_1)$ and M_1 are conditionally independent given C. Thus, under assumptions (MN.5b), assumption (MN.4) means that $f_{M_2(x', m_1')|C}\left(m_2 \left| c\right.\right)$ can be re-written as $f_{M_2|C,X,M_1}\left(m_2 \left| c, x', m_1'\right.\right)$. Under assumption (MC.2),

$E\left\{Y\left(1,m_1,m_2\right)-Y\left(0,m_1,m_2\right)\left|C=c\right.\right\}$ is identified using the g-computation formula, in a variant of (A.3) that does not marginalise over C:

$$
\begin{aligned}
&E\left\{Y\left(1,m_1,m_2\right)-Y\left(0,m_1,m_2\right)\left|C=c\right.\right\}= \\
&\qquad\int_{\mathcal{L}_1}\int_{\mathcal{L}_2}\left\{E\left(Y\left|C=c,X=1,L_1=l_1,M_1=m_1,L_2=l_2,M_2=m_2\right.\right)\right. \\
&\qquad\qquad\cdot f_{L_1|C,X}\left(l_1\left|c,1\right.\right)f_{L_2|C,X,L_1,M_1}\left(l_2\left|c,1,l_1,m_1\right.\right) \\
&\qquad\quad-E\left(Y\left|C=c,X=0,L_1=l_1,M_1=m_1,L_2=l_2,M_2=m_2\right.\right) \\
&\qquad\qquad\left.\cdot f_{L_1|C,X}\left(l_1\left|c,0\right.\right)f_{L_2|C,X,L_1,M_1}\left(l_2\left|c,0,l_1,m_1\right.\right)\right\}d\mu_{L_2}\left(l_2\right)d\mu_{L_1}\left(l_1\right). \quad \text{(A.38)}
\end{aligned}
$$

As before, the boxed quantity in (A.37) is not identified, and requires a stronger parametric model specification, or a sensitivity analysis, as discussed in Web Appendix J.

(b) identifying natural indirect effects through M_1 alone.

Each NIE_1 (see Table 1 in the main manuscript) is of the form

$$E\left\{Y\left(x,M_1(1),M_2(x',M_1(x''))\right)-Y\left(x,M_1(0),M_2(x',M_1(x''))\right)\right\}$$

for some x, x', x''. This can be re-written as:

$$
\begin{aligned}
&E\left\{Y\left(x, M_1(1), M_2(x', M_1(x''))\right) - Y\left(x, M_1(0), M_2(x', M_1(x''))\right)\right\} \\
&= \int_{\mathcal{C}} \int_{\mathcal{M}_1} \int_{\mathcal{M}_1} \int_{\mathcal{M}_1} \int_{\mathcal{M}_2} E\left\{Y\left(x, M_1(1), M_2(x', M_1(x''))\right)\right. \\
&- Y\left(x, M_1(0), M_2(x', M_1(x''))\right) \left| C = c, M_1(1) = m_1, M_1(0) = m_1', \right. \\
&\left. M_2(x', m_1'') = m_2, M_1(x'') = m_1''\right\} f_{M_2(x', m_1'')|C, M_1(x''), M_1(1), M_1(0)}\left(m_2 \left| c, m_1'', m_1, m_1'\right.\right) \\
&\cdot \left\{I\left(x'' = 1\right) I\left(m_1'' = m_1\right) + I\left(x'' = 0\right) I\left(m_1'' = m_1'\right)\right\} f_{M_1(1)|C, M_1(0)}\left(m_1 \left| c, m_1'\right.\right) \\
&\cdot f_{M_1(0)|C}\left(m_1' \left| c\right.\right) f_C(c)\, d\mu_{M_2}(m_2)\, d\mu_{M_1}(m_1'')\, d\mu_{M_1}(m_1')\, d\mu_{M_1}(m_1)\, d\mu_C(c) \\
&= \int_{\mathcal{C}} \int_{\mathcal{M}_1} \int_{\mathcal{M}_1} \int_{\mathcal{M}_1} \int_{\mathcal{M}_2} E\left\{Y\left(x, m_1, m_2\right) - Y\left(x, m_1', m_2\right) \left| C = c\right.\right\} \\
&\cdot f_{M_2(x', m_1'')|C}\left(m_2 \left| c\right.\right) \left\{I\left(x'' = 1\right) I\left(m_1'' = m_1\right) + I\left(x'' = 0\right) I\left(m_1'' = m_1'\right)\right\} \\
&\cdot f_{M_1(1)|C, M_1(0)}\left(m_1 \left| c, m_1'\right.\right) f_{M_1(0)|C}\left(m_1' \left| c\right.\right) f_C(c) \qquad \text{(A.39)} \\
&\cdot d\mu_{M_2}(m_2)\, d\mu_{M_1}(m_1'')\, d\mu_{M_1}(m_1')\, d\mu_{M_1}(m_1)\, d\mu_C(c) \qquad \text{(A.40)} \\
&= \int_{\mathcal{C}} \int_{\mathcal{M}_1} \int_{\mathcal{M}_1} \int_{\mathcal{M}_1} \int_{\mathcal{M}_2} E\left\{Y\left(x, m_1, m_2\right) - Y\left(x, m_1', m_2\right) \left| C = c\right.\right\} \\
&\cdot f_{M_2|C, X, M_1}\left(m_2 \left| c, x', m_1''\right.\right) \left\{I\left(x'' = 1\right) I\left(m_1'' = m_1\right) + I\left(x'' = 0\right) I\left(m_1'' = m_1'\right)\right\} \\
&\cdot \boxed{f_{M_1(1)|C, M_1(0)}\left(m_1 \left| c, m_1'\right.\right)} f_{M_1|C, X}\left(m_1' \left| c, x\right.\right) f_C(c) \\
&\cdot d\mu_{M_2}(m_2)\, d\mu_{M_1}(m_1'')\, d\mu_{M_1}(m_1')\, d\mu_{M_1}(m_1)\, d\mu_C(c). \qquad \text{(A.41)}
\end{aligned}
$$

Steps (A.40) and (A.41) follow from assumptions (MN.4) and (MN.5b) using the same arguments as given for the NDEs above. Under assumption (MC.2), $E\left\{Y\left(x, m_1, m_2\right) - Y\left(x, m_1', m_2\right) \left| C = c\right.\right\}$ is identified using the g-computation formula, in

an adaptation of (A.38):

$$
\begin{aligned}
&E\left\{Y\left(x,m_1,m_2\right)-Y\left(x,m_1',m_2\right)|C=c\right\}= \\
&\quad\int_{\mathcal{L}_1}\int_{\mathcal{L}_2}\left\{E\left(Y\,|C=c,X=x,L_1=l_1,M_1=m_1,L_2=l_2,M_2=m_2\right)\right. \\
&\qquad\cdot f_{L_2|C,X,L_1,M_1}\left(l_2\,|c,x,l_1,m_1\right) \\
&\qquad-E\left(Y\,|C=c,X=x,L_1=l_1,M_1=m_1',L_2=l_2,M_2=m_2\right) \\
&\qquad\left.\cdot f_{L_2|C,X,L_1,M_1}\left(l_2\,|c,x,l_1,m_1'\right)\right\}f_{L_1|C,X}\left(l_1\,|c,x\right)d\mu_{L_2}\left(l_2\right)d\mu_{L_1}\left(l_1\right).
\end{aligned}
$$

Again, the boxed quantity in (A.41) is not identified, and thus the approach discussed in Web Appendix J is needed.

(c) identifying natural indirect effects through M_2 alone.

Each NIE_2 (see Table 1 in the main manuscript) is of the form

$$E\left\{Y\left(x,M_1(x'),M_2(1,M_1(x''))\right)-Y\left(x,M_1(x'),M_2(0,M_1(x''))\right)\right\}$$

for some x,x',x''. This can be re-written as:

$$
\begin{aligned}
&E\left\{Y\left(x,M_1(x'),M_2(1,M_1(x''))\right)-Y\left(x,M_1(x'),M_2(0,M_1(x''))\right)\right\} \\
&=\int_{\mathcal{C}}\int_{\mathcal{M}_1}\int_{\mathcal{M}_1}\int_{\mathcal{M}_2}\int_{\mathcal{M}_2}E\left\{Y\left(x,M_1(x'),M_2(1,M_1(x''))\right)\right. \\
&\qquad-Y\left(x,M_1(x'),M_2(0,M_1(x''))\right)|C=c,M_1(x')=m_1,M_2(1,m_1')=m_2 \\
&\qquad\left.M_2(0,m_1')=m_2',M_1(x'')=m_1'\right\} \\
&\qquad\cdot f_{M_2(1,m_1')\left|C,M_2(0,m_1'),M_1(x''),M_1(x')\right.}\left(m_2\,|c,m_2',m_1',m_1\right) \\
&\qquad\cdot f_{M_2(0,m_1')|C,M_1(x''),M_1(x')}\left(m_2'\,|c,m_1',m_1\right)f_{M_1(x'')|C,M_1(x')}\left(m_1'\,|c,m_1\right) \\
&\qquad\cdot f_{M_1(x')|C}\left(m_1\,|c\right)f_C\left(c\right)d\mu_{M_2}\left(m_2'\right)d\mu_{M_2}\left(m_2\right)d\mu_{M_1}\left(m_1'\right)d\mu_{M_1}\left(m_1\right)d\mu_C\left(c\right)
\end{aligned}
$$

$$= \int_{\mathcal{C}} \int_{\mathcal{M}_1} \int_{\mathcal{M}_1} \int_{\mathcal{M}_2} \int_{\mathcal{M}_2} E\left\{Y\left(x, m_1, m_2\right) - Y\left(x, m_1, m_2'\right) \left| C = c\right.\right\}$$

$$\cdot f_{M_2(1,m_1')|C,M_2(0,m_1')}\left(m_2 \left| c, m_2'\right.\right) f_{M_2(0,m_1')|C}\left(m_2' \left| c\right.\right)$$

$$\cdot f_{M_1(x'')|C,M_1(x')}\left(m_1' \left| c, m_1\right.\right) f_{M_1(x')|C}\left(m_1 \left| c\right.\right) f_C\left(c\right)$$

$$\cdot d\mu_{M_2}\left(m_2'\right) d\mu_{M_2}\left(m_2\right) d\mu_{M_1}\left(m_1'\right) d\mu_{M_1}\left(m_1\right) d\mu_C\left(c\right) \qquad \text{(A.42)}$$

$$= \int_{\mathcal{C}} \int_{\mathcal{M}_1} \int_{\mathcal{M}_1} \int_{\mathcal{M}_2} \int_{\mathcal{M}_2} E\left\{Y\left(x, m_1, m_2\right) - Y\left(x, m_1, m_2'\right) \left| C = c\right.\right\}$$

$$\cdot \boxed{f_{M_2(1,m_1')|C,M_2(0,m_1')}\left(m_2 \left| c, m_2'\right.\right)} f_{M_2|C,X,M_1}\left(m_2' \left| c, 0, m_1'\right.\right)$$

$$\cdot \boxed{f_{M_1(x'')|C,M_1(x')}\left(m_1' \left| c, m_1\right.\right)} f_{M_1|C,X}\left(m_1 \left| c, x'\right.\right) f_C\left(c\right)$$

$$\cdot d\mu_{M_2}\left(m_2'\right) d\mu_{M_2}\left(m_2\right) d\mu_{M_1}\left(m_1'\right) d\mu_{M_1}\left(m_1\right) d\mu_C\left(c\right). \qquad \text{(A.43)}$$

Steps (A.42) and (A.43) follow from assumptions (MN.4) and (MN.5b) using the same arguments as given for the NDEs above. Under assumption (MC.2), $E\left\{Y\left(x, m_1, m_2\right) - Y\left(x, m_1, m_2'\right) \left| C = c\right.\right\}$ is identified as follows:

$$E\left\{Y\left(x, m_1, m_2\right) - Y\left(x, m_1, m_2'\right) \left| C = c\right.\right\} =$$

$$\int_{\mathcal{L}_1} \int_{\mathcal{L}_2} \left\{E\left(Y \left| C = c, X = x, L_1 = l_1, M_1 = m_1, L_2 = l_2, M_2 = m_2\right.\right)\right.$$

$$\left. - E\left(Y \left| C = c, X = x, L_1 = l_1, M_1 = m_1, L_2 = l_2, M_2 = m_2'\right.\right)\right\}$$

$$\cdot f_{L_2|C,X,L_1,M_1}\left(l_2 \left| c, x, l_1, m_1\right.\right) f_{L_1|C,X}\left(l_1 \left| c, x\right.\right) d\mu_{L_2}\left(l_2\right) d\mu_{L_1}\left(l_1\right).$$

There are now two boxed quantities in (A.43) that are not identified. The approach discussed in Web Appendix J would need to be extended to incorporate the joint cross-world distribution of $M_2(x, m_1)$ for different x, as well as the joint cross-world distribution of $M_1(x)$ for different x. This can be done in exactly the way described in Web Appendix J but would now involve two sensitivity parameters rather than one.

Web Appendix M: More details on the data analysis

In this appendix more details of the data analysis (see section 6 of the main manuscript) are given. Web Table 3 gives some descriptive statistics. In Web Table 4, the Akaike Information Criterion (AIC) for various choices of the associational models is given; out of these, the model with the highest AIC was chosen. The estimated coefficients (and SEs) for the final chosen model is shown in Web Table 5.

[Web Table 3 about here.]

[Web Table 4 about here.]

[Web Table 5 about here.]

Web Appendix N: Plots for the sensitivity analysis varying the value of κ

In this section we include several plots based on the analysis of the data from the Izhevsk Family Study (see Section 6 of the main manuscript), mainly focussing on the sensitivity parameter κ.

The general conclusion is that the results here are not very sensitive to the value of κ. The final figures (16 to 19) confirm that those effects whose estimation did not require knowledge of κ are indeed insensitive to the value of κ. Of note is the fact that the summary effects are less sensitive to κ than most of the other effects.

[Web Figure 6 about here.]

[Web Figure 7 about here.]

[Web Figure 8 about here.]

[Web Figure 9 about here.]

[Web Figure 10 about here.]

[Web Figure 11 about here.]

[Web Figure 12 about here.]

[Web Figure 13 about here.]

[Web Figure 14 about here.]

[Web Figure 15 about here.]

[Web Figure 16 about here.]

[Web Figure 17 about here.]

[Web Figure 18 about here.]

[Web Figure 19 about here.]

Web Appendix P: Assumptions for identifying path-specific effects with n causally-ordered mediators

In this section we extend assumptions (MC.2), (MN.4), (MN.5) and (MN.5b) to (nMC.2), (nMN.4), (nMN.5) and (nMN.5b), respectively, extending from two mediators to n. The arguments that show that each path-specific effect can then be identified (up to $n-1$ sensitivity parameters) are omitted, but are direct extensions of what was given in Section 4 of the main manuscript and the Web Appendices references therein.

Assumption (nMC.2).

There exist measured sets of variables, C, L_1, $\ldots$, L_n such that, for $x = 0,1$ and $\forall c, l_1, \ldots, l_n, m_1, \ldots, m_n$:

$$Y(x, m_1, \ldots, m_n) \perp\!\!\!\perp X \,|C = c\,,$$

$$Y(x, m_1, \ldots, m_n) \perp\!\!\!\perp M_1 \,|C = c, X = x, L_1 = l_1\,,$$

and

$$Y(x, m_1, \ldots, m_n) \perp\!\!\!\perp M_j \,|C = c, X = x, M_1 = m_1, \ldots, M_{j-1} = m_{j-1}, L_1 = l_1, \ldots, L_j = l_j \quad j = 2, \ldots, n.$$

$\square$

Assumption (nMN.4).

For $x = 0, 1$ and $\forall c$:

$$M_1(x) \perp\!\!\!\perp X \,|C = c\,,$$

for $x = 0, 1$, $j = 2, \ldots, n$, and $\forall c, m_1, \ldots, m_{n-1}$:

$$M_j(x, m_1, \ldots, m_{j-1}) \perp\!\!\!\perp X \,|C = c$$

and for $x = 0, 1$, $j = 2, \ldots, n$, $k = 1, \ldots, j-1$ and $\forall c, l_1, \ldots, l_{n-1}, m_1, \ldots, m_{n-1}$:

$$M_j(x, m_1, \ldots, m_{j-1}) \perp\!\!\!\perp M_k \,|C = c, X = x, M_1 = m_1, \ldots, M_{k-1} = m_{k-1}, L_1 = l_1, \ldots, L_k = l_k$$

$\square$

Assumption (nMN.5). $\forall c, l_1$:

$$Y(x, m_1, \ldots, m_n) \perp\!\!\!\perp \{M_1(x^{\cdot})\} \,|C = c, L_1 = l_1\,,$$

for $j = 2, \ldots, n$, $\forall c, l_1, \ldots, l_n, m_1, \ldots, m_n$:

$$Y(x, m_1, \ldots, m_n) \perp\!\!\!\perp \left\{M_j\left(x^{\cdot}, m_1^{\cdot}, \ldots, m_{j-1}^{\cdot}\right)\right\} |C = c, L_1 = l_1, \ldots, L_j = l_j, \{M_1(x^{\cdot})\} = m_1, \ldots,$$
$$\left\{M_{j-1}\left(x^{\cdot}, m_1^{\cdot}, \ldots, m_{j-2}^{\cdot}\right) = m_{j-1}\right\},$$

and for $j = 2, \ldots, n$, $k = 1, \ldots, j-1$, $\forall c, l_1, \ldots, l_n, m_1, \ldots, m_n$:

$$M_j(x, m_1, \ldots, m_{j-1}) \perp\!\!\!\perp \left\{M_k\left(x^{\cdot}, m_1^{\cdot}, \ldots, m_{k-1}^{\cdot}\right)\right\} |C = c, L_1 = l_1, \ldots, L_k = l_k, \{M_1(x^{\cdot}) = m_1\}, \ldots,$$
$$\left\{M_{k-1}\left(x^{\cdot}, m_1^{\cdot}, \ldots, m_{k-2}^{\cdot}\right) = m_{k-1}\right\}$$

$\square$

Assumption (nMN.5b).

$$E[Y(1, m_1, \ldots, m_n) - Y(0, m_1, \ldots, m_n) \,|\{M_1(x^{\cdot})\}, C]$$
$$= E[Y(1, m_1, \ldots, m_n) - Y(0, m_1, \ldots, m_n) \,|C]\,,$$

$$E\left[Y\left(x,m_1,m_2,\ldots,m_n\right)-Y\left(x,m_1',m_2,\ldots,m_n\right)\left|\left\{M_1\left(x^{\cdot}\right)\right\},C\right.\right]$$
$$=E\left[Y\left(x,m_1,m_2,\ldots,m_n\right)-Y\left(x,m_1',m_2,\ldots,m_n\right)\left|C\right.\right],$$

...

$$E\left[Y\left(x,m_1,\ldots,m_{n-1},m_n\right)-Y\left(x,m_1,\ldots,m_{n-1},m_n'\right)\left|\left\{M_1\left(x^{\cdot}\right)\right\},C\right.\right]$$
$$=E\left[Y\left(x,m_1,\ldots,m_{n-1},m_n\right)-Y\left(x,m_1,\ldots,m_{n-1},m_n'\right)\left|C\right.\right],$$

for $j=2,\ldots,n$:

$$E\left[Y\left(1,m_1,\ldots,m_n\right)-Y\left(0,m_1,\ldots,m_n\right)\right|$$
$$\left\{M_1\left(x^{\cdot}\right)\right\},\ldots,\left\{M_j\left(x^{\cdot},m_1^{\cdot},\ldots,m_{j-1}^{\cdot}\right),C\right\}]$$
$$=E\left[Y\left(1,m_1,\ldots,m_n\right)-Y\left(0,m_1,\ldots,m_n\right)\right|$$
$$\left\{M_1\left(x^{\cdot}\right)\right\},\ldots,\left\{M_{j-1}\left(x^{\cdot},m_1^{\cdot},\ldots,m_{j-2}^{\cdot}\right),C\right\}],$$

$$E\left[Y\left(x,m_1,m_2,\ldots,m_n\right)-Y\left(x,m_1',m_2,\ldots,m_n\right)\right|$$
$$\left\{M_1\left(x^{\cdot}\right)\right\},\ldots,\left\{M_j\left(x^{\cdot},m_1^{\cdot},\ldots,m_{j-1}^{\cdot}\right),C\right\}]$$
$$=E\left[Y\left(x,m_1,m_2,\ldots,m_n\right)-Y\left(x,m_1',m_2,\ldots,m_n\right)\right|$$
$$\left\{M_1\left(x^{\cdot}\right)\right\},\ldots,\left\{M_{j-1}\left(x^{\cdot},m_1^{\cdot},\ldots,m_{j-2}^{\cdot}\right),C\right\}],$$

$$E\left[Y\left(x,m_1,\ldots,m_{n-1},m_n\right)-Y\left(x,m_1,\ldots,m_{n-1},m_n'\right)\right|$$
$$\left\{M_1\left(x^{\cdot}\right)\right\},\ldots,\left\{M_j\left(x^{\cdot},m_1^{\cdot},\ldots,m_{j-1}^{\cdot}\right),C\right\}]$$
$$=E\left[Y\left(x,m_1,\ldots,m_{n-1},m_n\right)-Y\left(x,m_1,\ldots,m_{n-1},m_n'\right)\right|$$
$$\left\{M_1\left(x^{\cdot}\right)\right\},\ldots,\left\{M_{j-1}\left(x^{\cdot},m_1^{\cdot},\ldots,m_{j-2}^{\cdot}\right),C\right\}],$$

for $j=2,\ldots,n$, $k=1,\ldots,j-1$:

$$M_j\left(x,m_1,\ldots,m_{j-1}\right)\perp\!\!\!\perp$$
$$\left\{M_k\left(x^{\cdot},m_1^{\cdot},\ldots,m_{k-1}^{\cdot}\right)\right\}\left|C,\left\{M_1\left(x^{\cdot}\right)\right\},\ldots,\left\{M_{k-1}\left(x^{\cdot},m_1^{\cdot},\ldots,m_{k-2}^{\cdot}\right)\right\}\right.$$

and for $j = 2, \ldots, n$:

$$M_j\left(x, m_1, \ldots, m_{j-1}\right) \perp\!\!\!\perp \left\{M_{j-1}\left(x^{\cdot}, m_1^{\cdot}, \ldots, m_{j-2}^{\cdot}\right)\right\} \left| C, \left[\left\{M_j\left(x^{\cdot}, m_1^{\cdot}, \ldots, m_{j-1}^{\cdot}\right)\right\} \setminus M_j\left(x, m_1, \ldots, m_{j-1}\right)\right]\right.$$

□

References

Albert, J. M. and Nelson, S. (2011). Generalized causal mediation analysis. *Biometrics* **67,** 1028–1038.

Hafeman, D. and VanderWeele, T. (2011). Alternative assumptions for the identification of direct and indirect effects. *Epidemiology* **22,** 753–764.

Imai, K. and Yamamoto, T. (2013). Identification and sensitivity analysis for multiple causal mechanisms: revisiting evidence from framing experiments. *Political Analysis* **21,** 141.

Kuha, J. and Golthorpe, J. H. (2010). Path analysis for discrete variables: the role of education in social mobility. *Journal of the Royal Statistical Society, Series A* **173,** 351–369.

Pearl, J. (1995). Causal diagrams for empirical research. *Biometrika* **82,** 669–709.

Petersen, M. L., Sinisi, S. E., and van der Laan, M. J. (2006). Estimation of direct causal effects. *Epidemiology* **17,** 276–284.

Robins, J. and Richardson, T. (2011). Alternative graphical causal models and the identification of direct effects. In: *Causality and Psychopathology: Finding the Determinants of Disorders and Their Cures,* P. Shrout, Editor. Oxford University Press. .

Robins, J., Richardson, T., and Spirtes, P. (2009). On identfication and inference for direct effects. *Unpublished manuscript* .

Robins, J. M. (1986). A new approach to causal inference in mortality studies with a

sustained exposure period — application to control of the healthy worker survivor effect. *Mathematical Modelling* **7,** 1393–1512.

Roy, J., Hogan, J. W., and Marcus, B. H. (2008). Principal stratification with predictors of compliance for randomized trials with 2 active treatments. *Biostatistics* **9,** 277–289.

Shpitser, I. and Pearl, J. (2008). Complete identification methods for the causal hierarchy. *Journal of Machine Learning Research* **9,** 1941–1979.

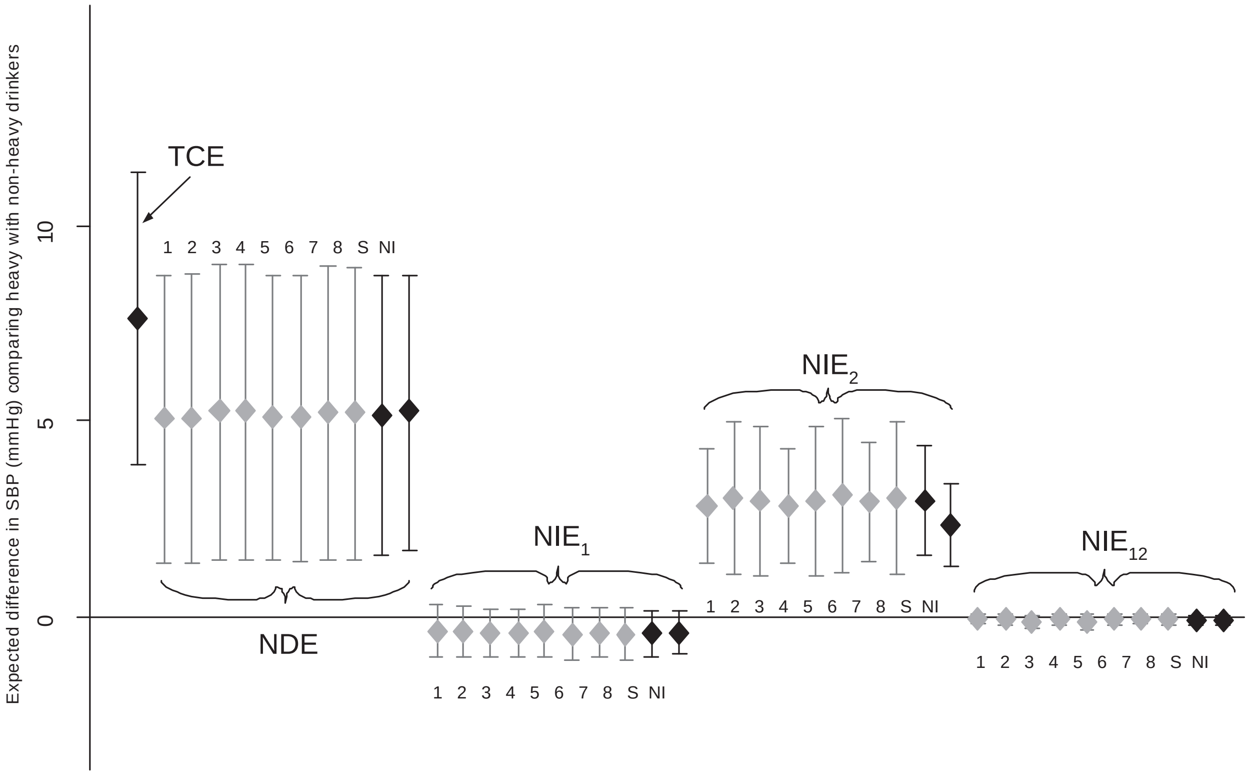

Figure 1. With $\kappa = 1$ (perfect correlation between $M_1(0)$ and $M_1(1)$ given C), estimates and 95% confidence intervals for the Total Causal Effect (TCE), followed by each of the eight versions of the Direct Effect (NDE), Indirect Effect through BMI alone (NIE_1), Indirect Effect through GGT alone (NIE_2) and Indirect Effect through both BMI and GGT (NIE_{12}). For all four path-specific effects, the eight versions are given (from left to right) in the following order: 000, 100, 010, 001, 110, 101, 011 and 111. The estimates (and 95% CIs) of the summary direct and indirect effects (labelled 'S') and the direct and indirect effects from a standard linear structural equation model assuming no interactions (labelled 'NI') are also shown.

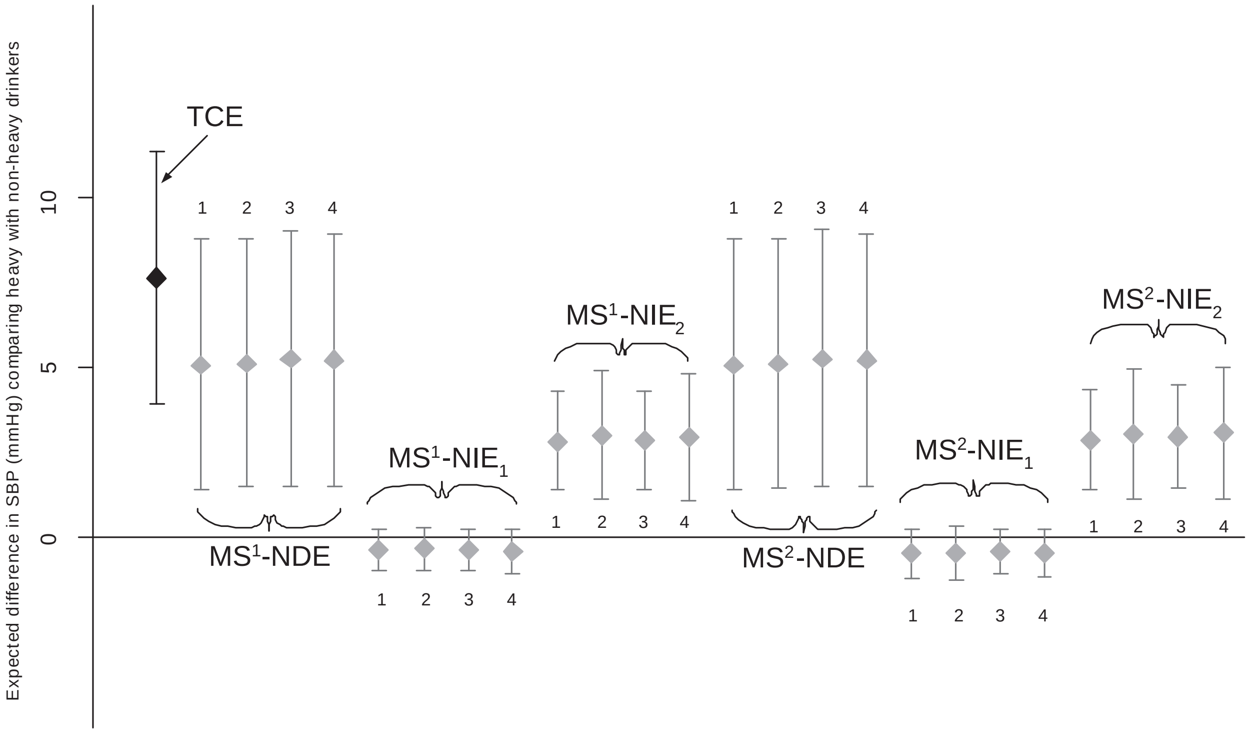

Figure 2. With $\kappa = 1$ (perfect correlation between $M_1(0)$ and $M_1(1)$ given C), estimates and 95% confidence intervals for the Total Causal Effect (TCE), followed by each of the four versions of the mediator-specific direct effect (NDE), indirect effect through BMI (NIE_1) and indirect effect through GGT (NIE_2). The two ways of defining these mediator-specific effects are given, using the superscripts 1 and 2, respectively. The four versions are given (from left to right) in the following order: 00, 10, 01 and 11.

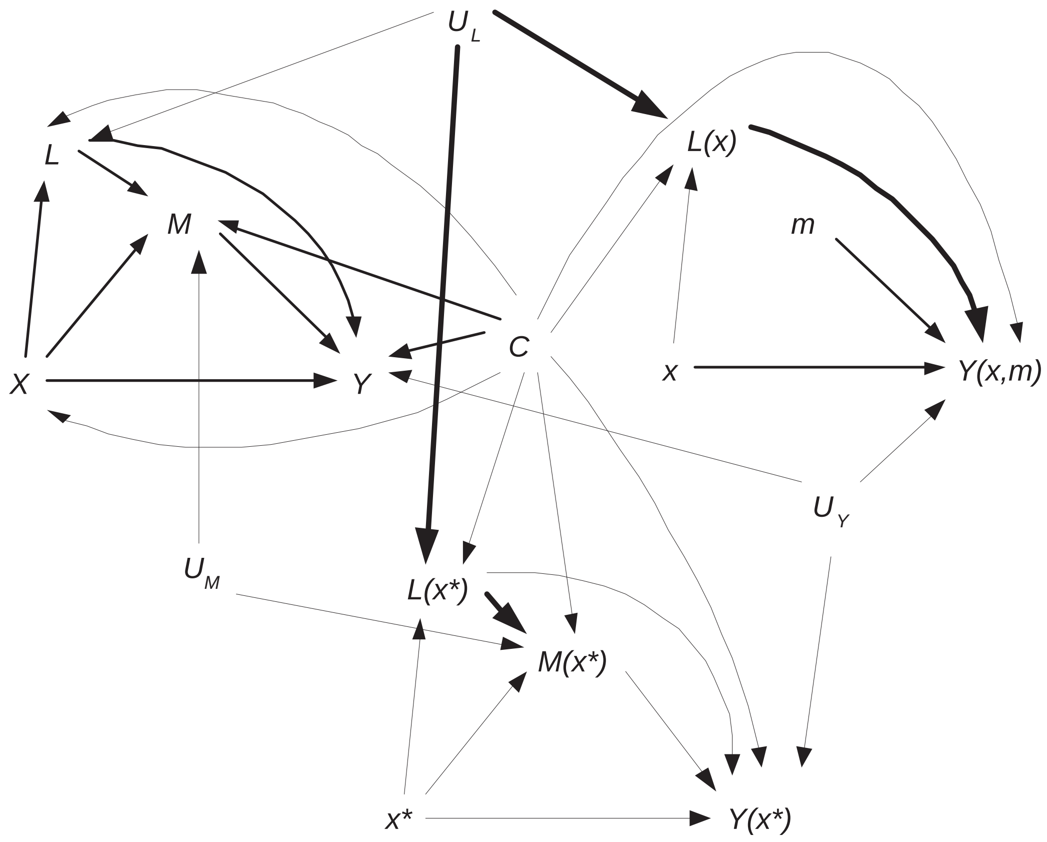

Figure 3. An illustration that $Y(x,m)$ and $M(x^*)$ are not in general conditionally independent given C and L whenever L is affected by X. The path shown in bold is not blocked by (C,L).

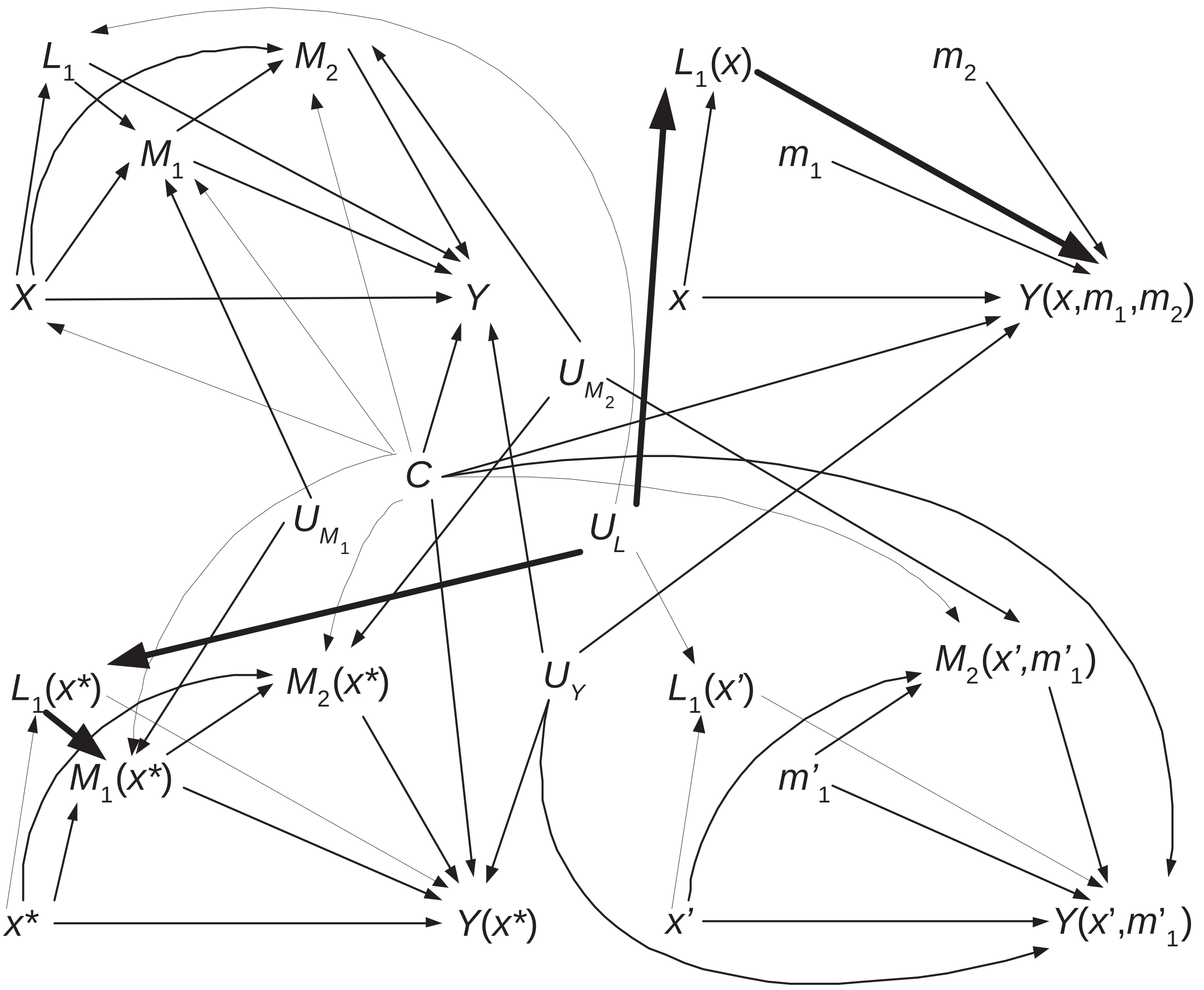

Figure 4. An illustration that $Y(x, m_1, m_2)$ and $M_1(x^*)$ are not in general conditionally independent given C and L_1 whenever L_1 is a common cause of M_1 and Y and is affected by X. The path shown in bold is not blocked by (C, L_1).

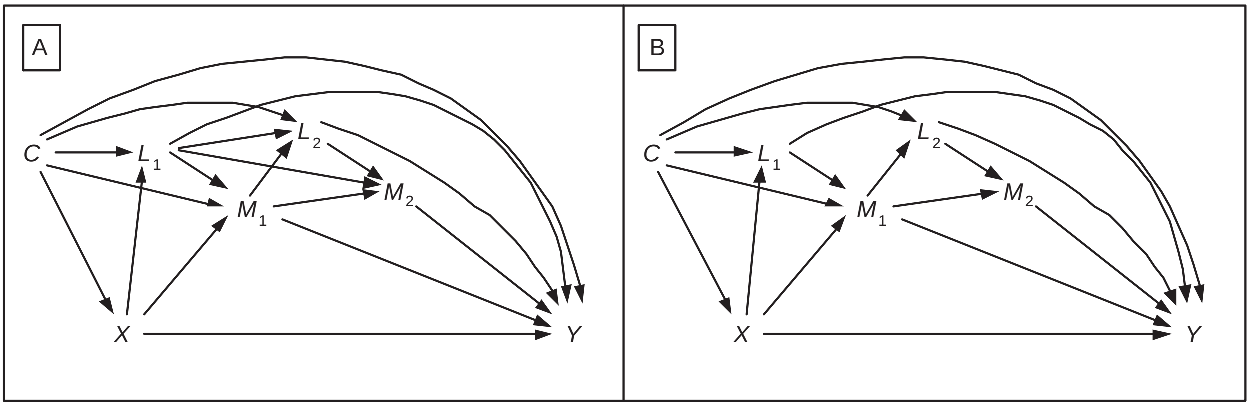

Figure 5. A. The general pattern of intermediate confounding, and B. the restricted pattern of intermediate confounding permitted when assumption (MN.7a) is relaxed to assumption (MN.7b).

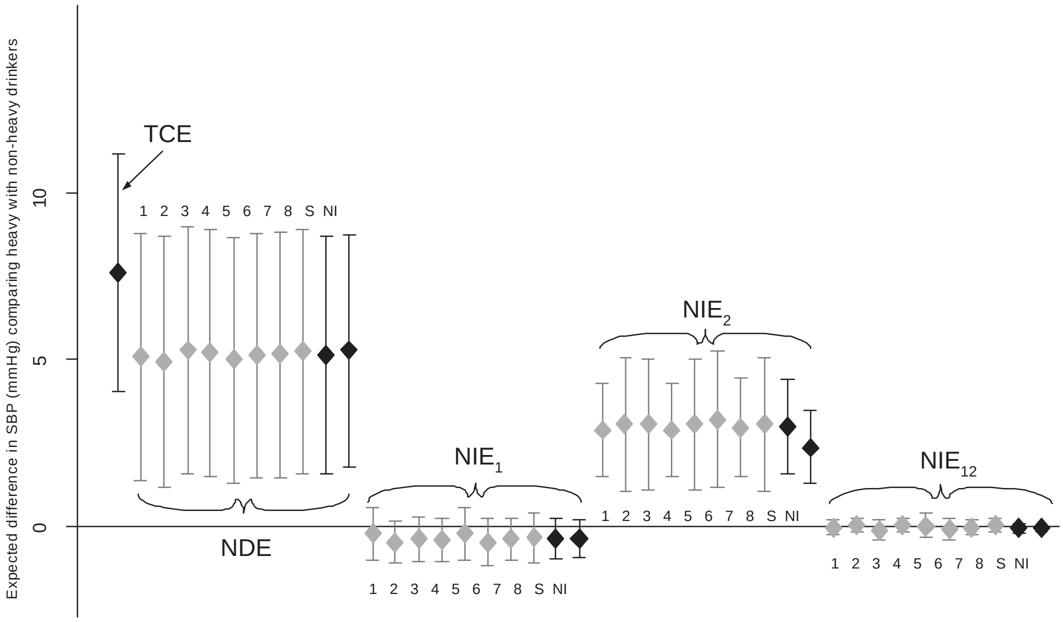

Figure 6. The same as Figure 1 in the Web Appendix, with $\kappa = 0.5$.

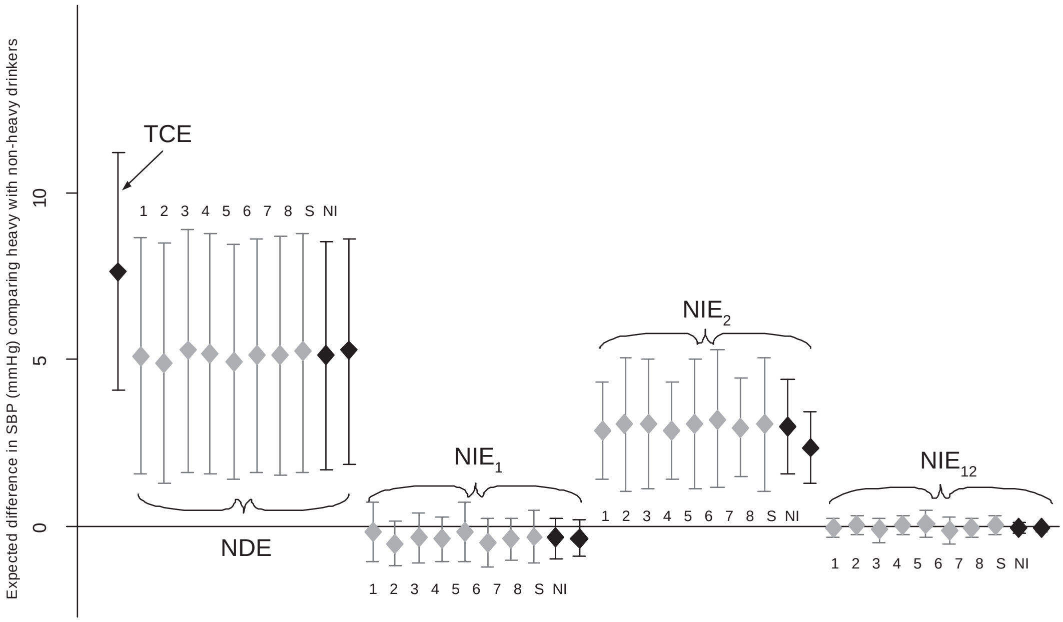

Figure 7. The same as Figure 1 in the Web Appendix, with $\kappa = 0$.

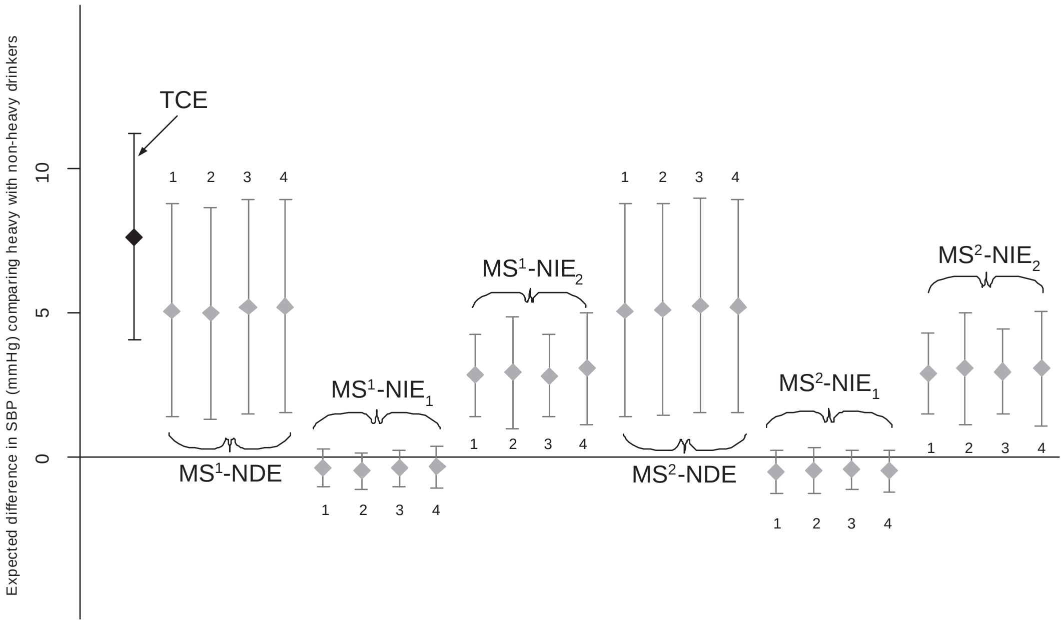

Figure 8. The same as Figure 4 in the main manuscript, with $\kappa = 0.5$.

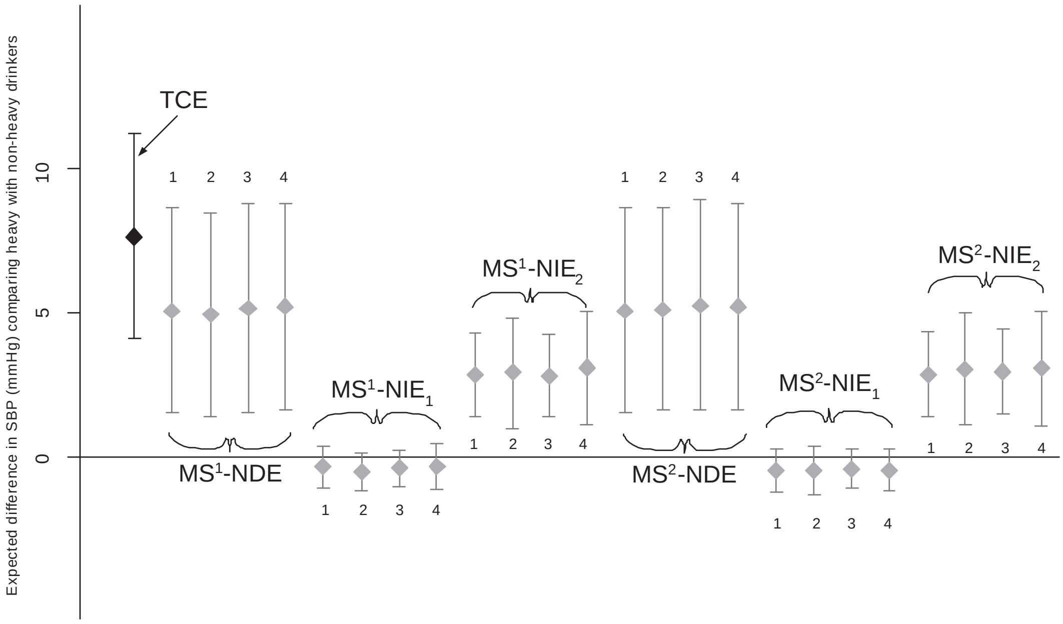

Figure 9. The same as Figure 4 in the main manuscript, with $\kappa = 0$.

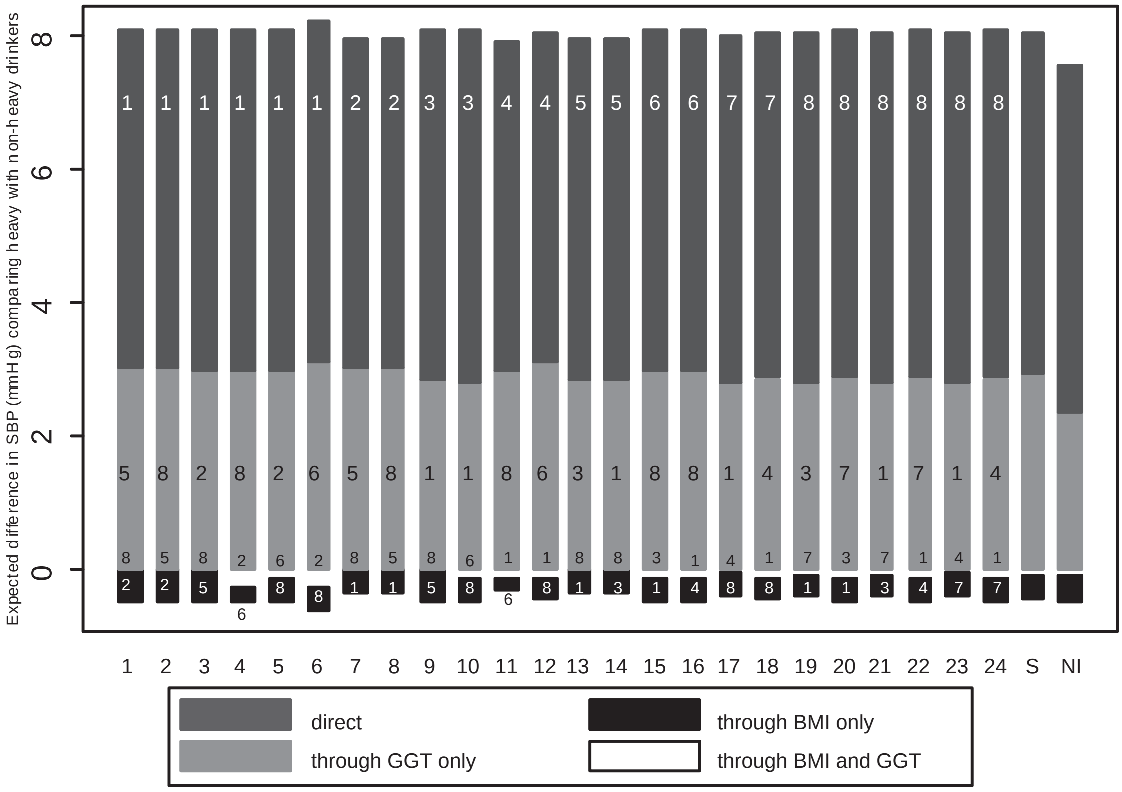

Figure 10. The same as Figure 2 in the Web Appendix but with $\kappa = 0.5$.

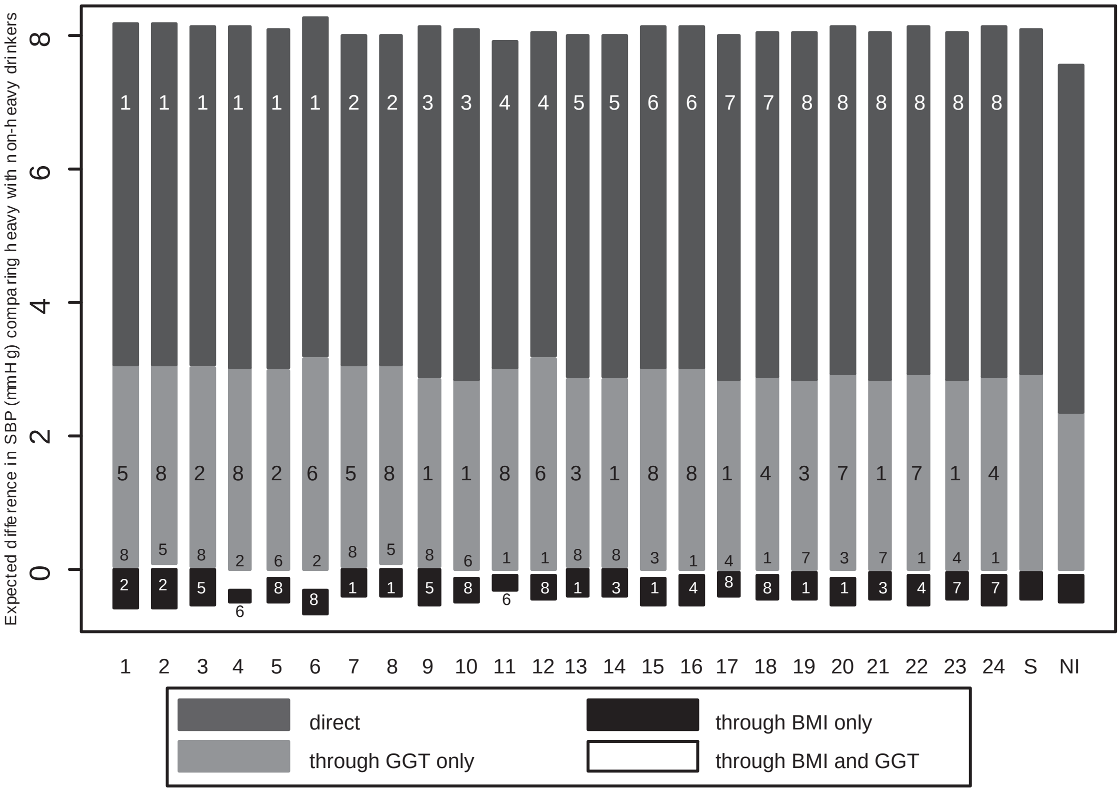

Figure 11. The same as Figure 2 in the Web Appendix but with $\kappa = 0$.

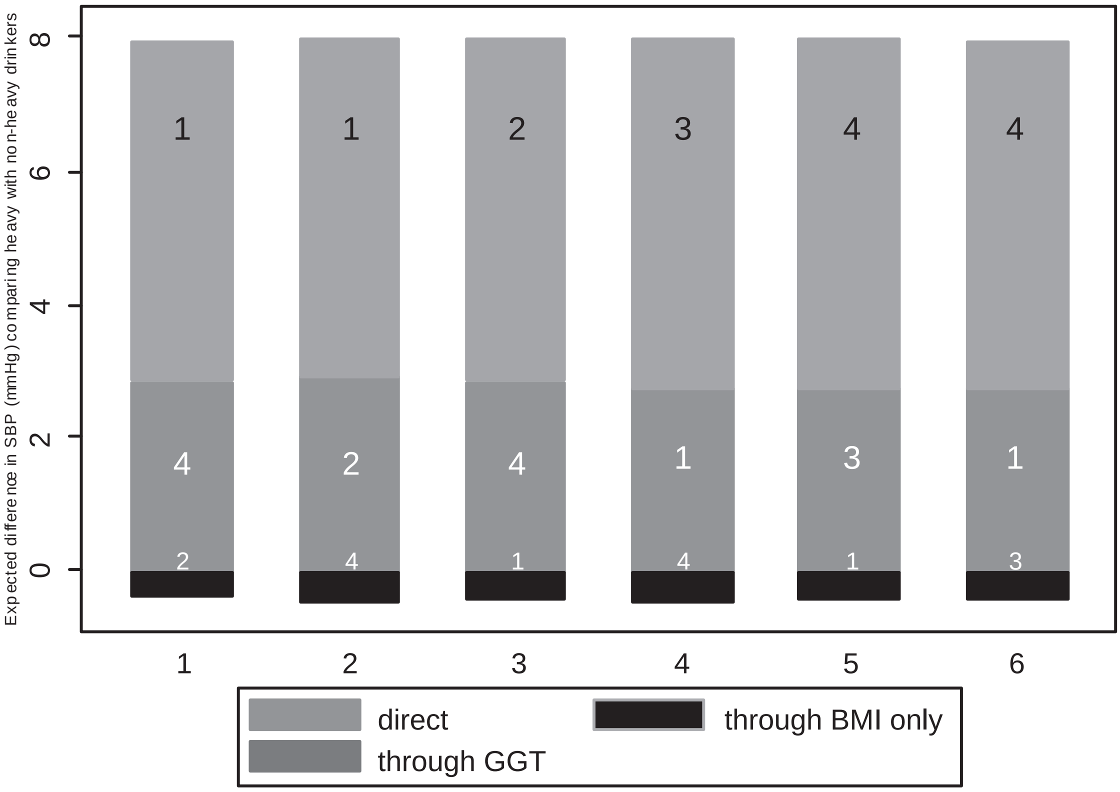

Figure 12. Similar to Figure 2 in the Web Appendix but showing the coarser decompositions into mediator-specific effects, according to the first definition of MS effects, with $\kappa = 1$. The numbers superimposed on the bars represent the code for that effect type (as defined in the caption of Table 1 in the Web Appendix). The numbers along the x-axis represent the decomposition number, also defined in Table 1 in the Web Appendix.

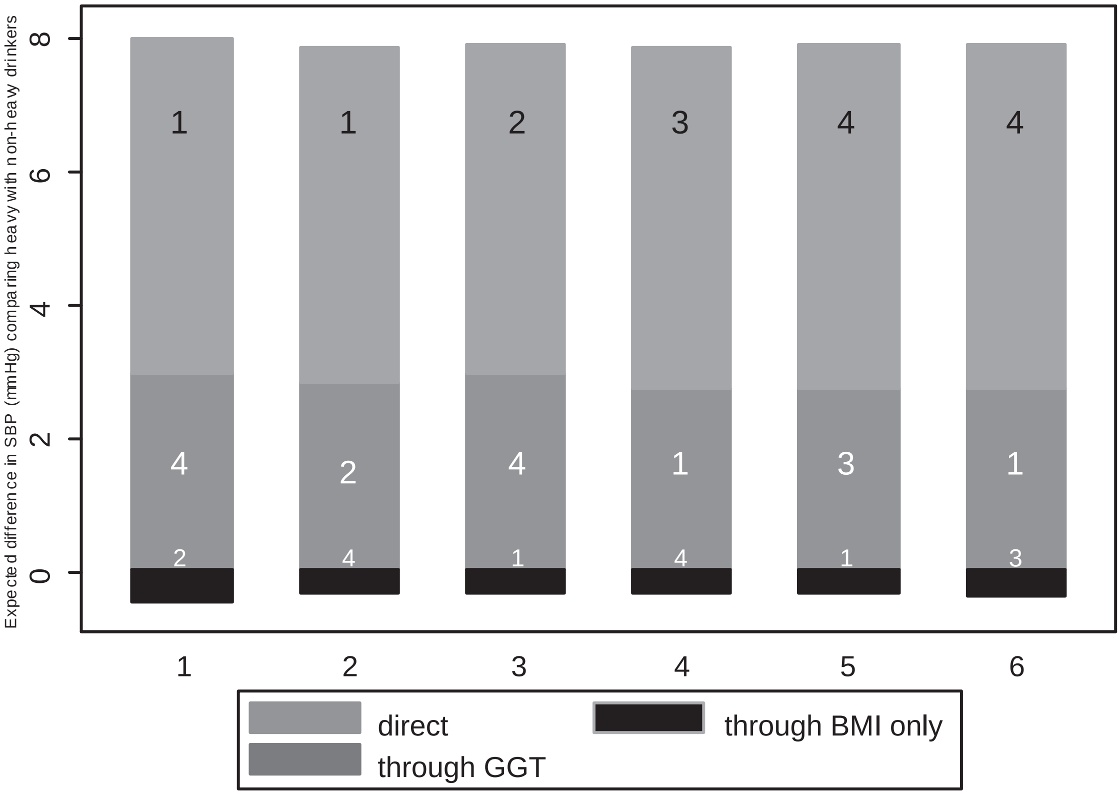

Figure 13. The same as Figure 12 in the Web Appendix but with $\kappa = 0.5$.

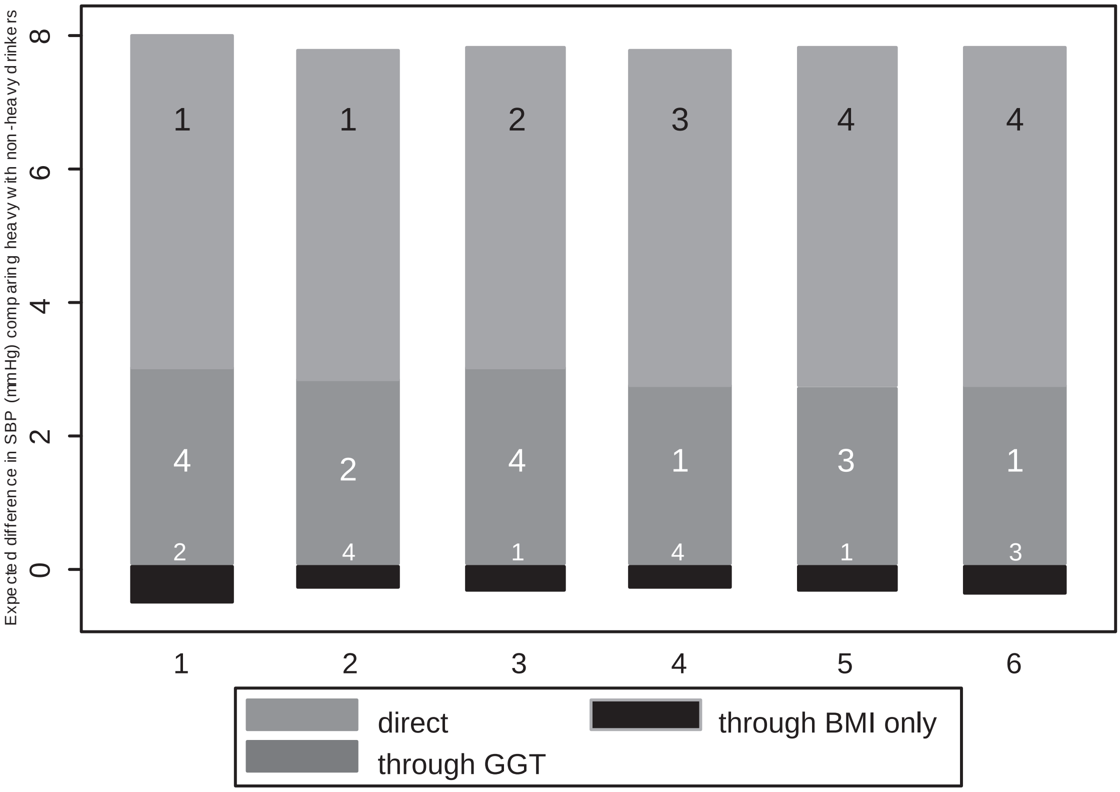

Figure 14. The same as Figure 12 in the Web Appendix but with $\kappa = 0$.

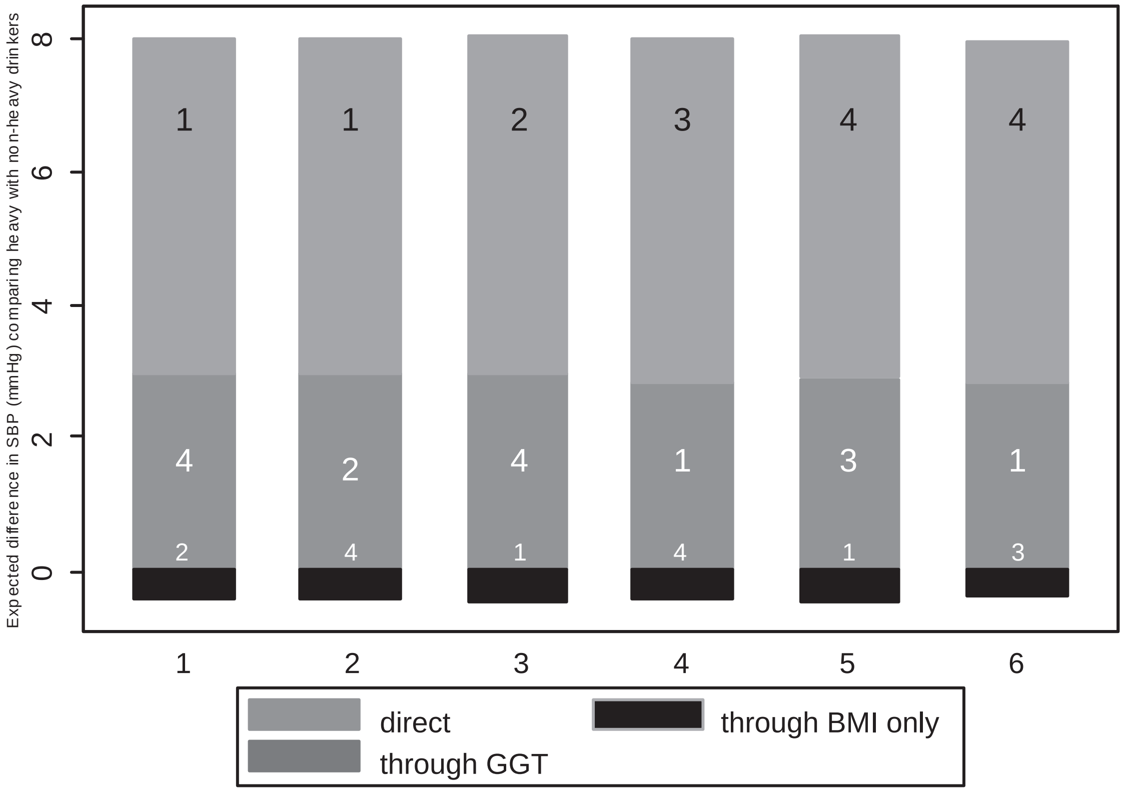

Figure 15. Similar to Figure 12 in the Web Appendix but using the second definition of a mediator-specific effect. These effects do not depend on κ.

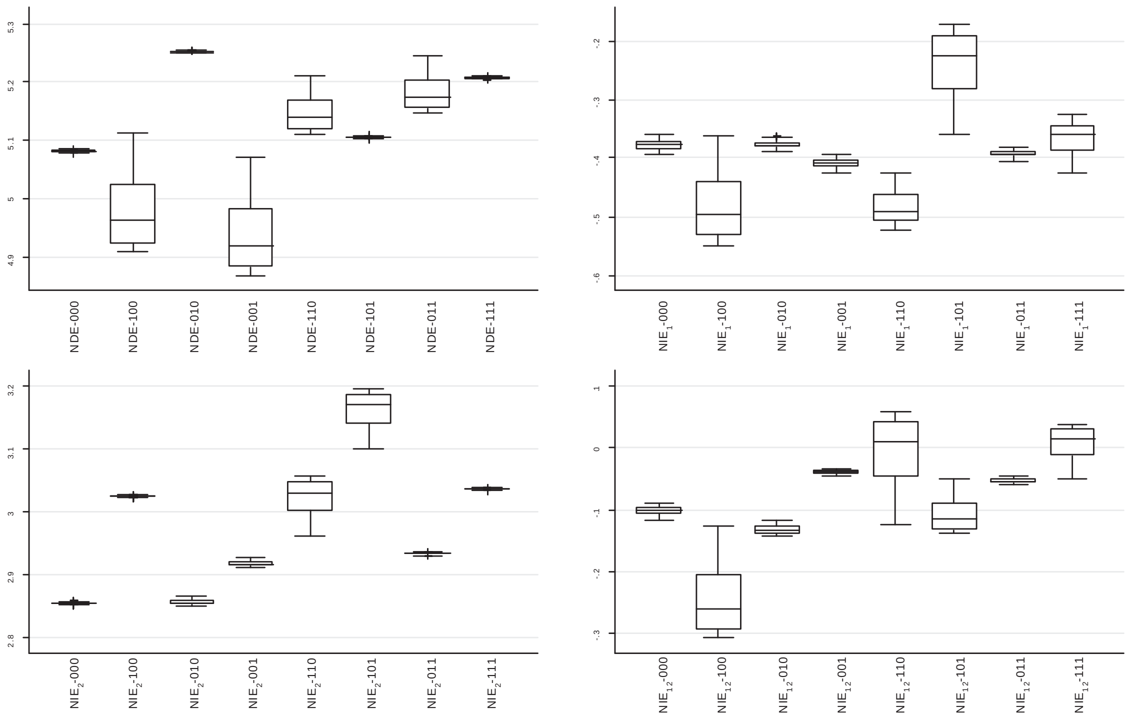

Figure 16. Box plots showing all 32 effects from Table 1 in the main manuscript estimated on the Izhevsk study data for different values of κ from 0 to 1 (increasing in increments of 0.001).

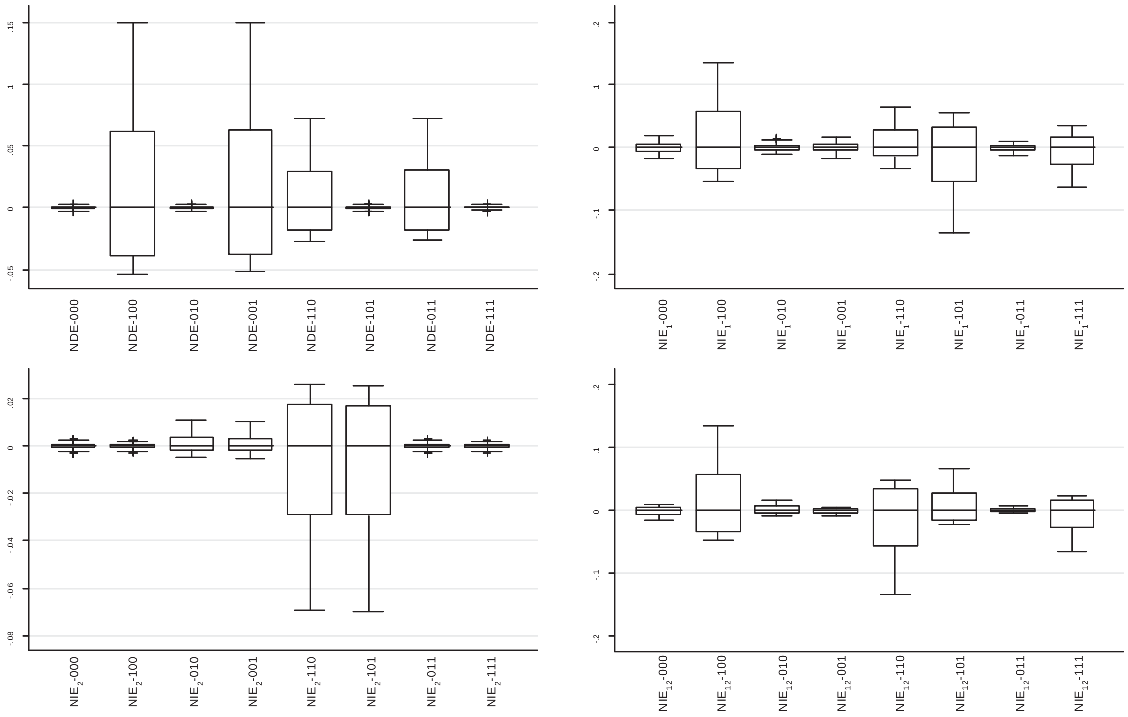

Figure 17. The same as Figure 16 in the Web Appendix but with each box plot centered at its median, so that the variability across effects can be more easily assessed.

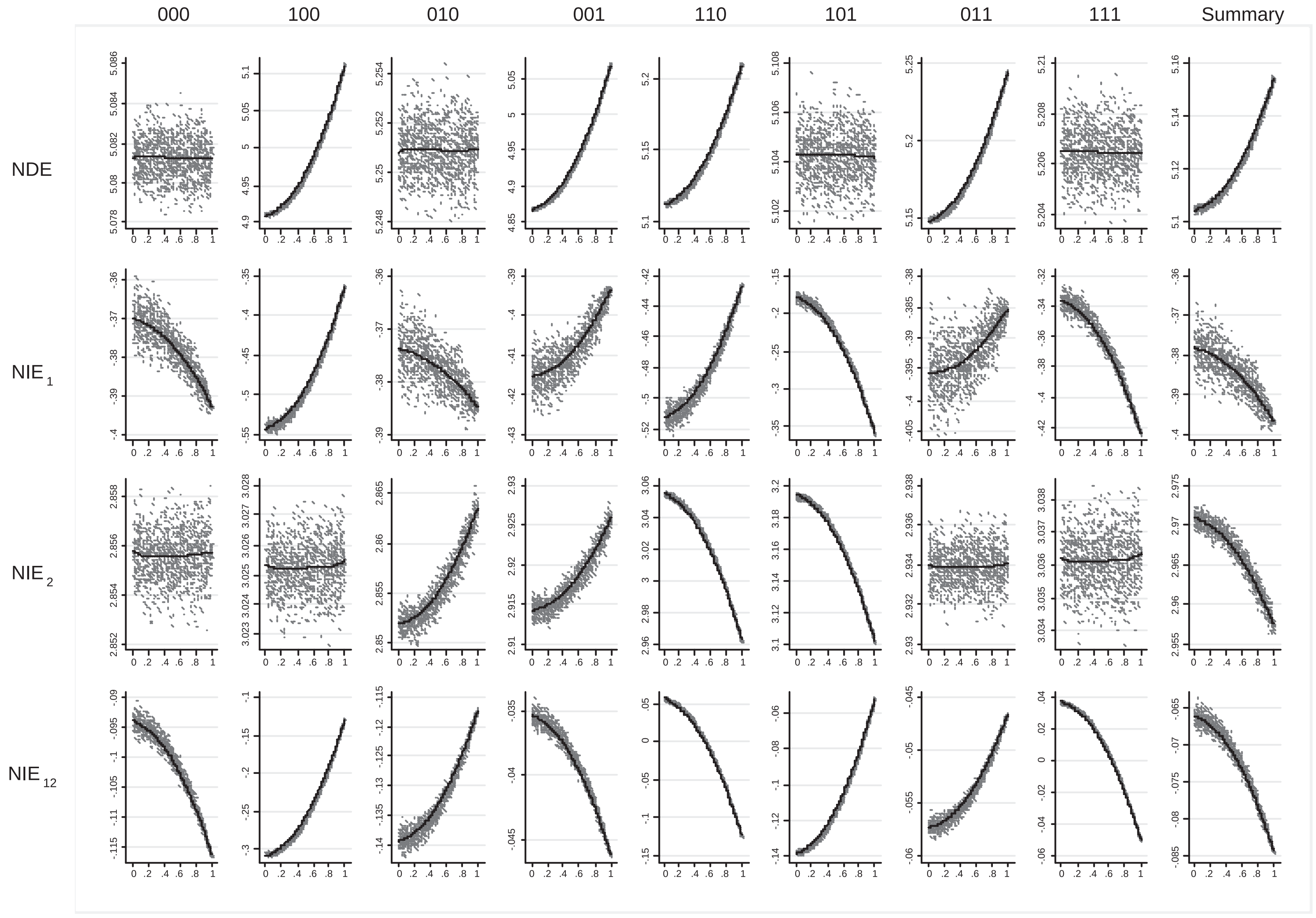

Figure 18. For each of the 32 effects (Table 1 in the main manuscript) estimated on the Izhevsk study data, a plot of the estimate against κ, with a lowess smoother. Also included, in the final column, is each of the summary path-specific effects.

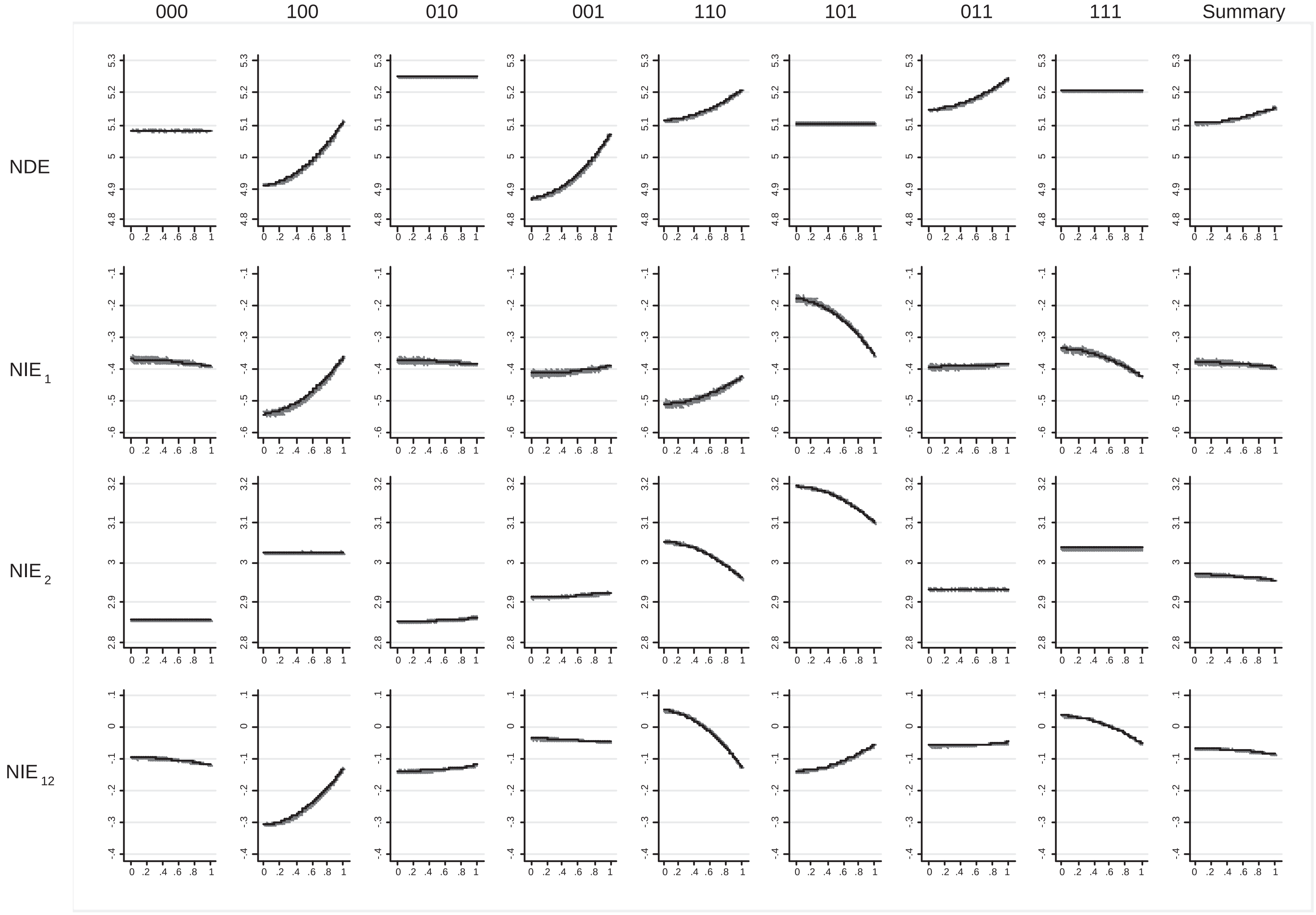

Figure 19. The same as Figure 18 in the Web Appendix except that the y-axis scale is the same for all columns in the same row, so that a comparison of sensitivity to κ can be made.

Table 1

All 6 possible decompositions of the total causal effect (TCE) into a direct effect (DE), an indirect effect via M_1 (IE_1) and an indirect effect via M_2 (IE_2) when M_1 and M_2 are not causally ordered. In each decomposition, there is one level-0 effect, one level-1 effect, and one level-2 effect. The definitions of each of these effects is given in Table 3 of the main manuscript. In columns 2–4, the effect types are labelled: 1=00, 2=10, 3=01, and 4=11.

Decomposition	Effect type			TCE =
	NDE	NIE_1	NIE_2	
1	1	2	4	NDE-00 + NIE_1-10 + NIE_2-11
2	1	4	2	NDE-00 + NIE_1-11 + NIE_2-10
3	2	1	4	NDE-10 + NIE_1-00 + NIE_2-11
4	3	4	1	NDE-01 + NIE_1-11 + NIE_2-00
5	4	1	3	NDE-11 + NIE_1-00 + NIE_2-01
6	4	3	1	NDE-11 + NIE_1-01 + NIE_2-00

Table 2

A summary of the comparison between the estimands defined and identified in the main manuscript versus those defined and identified in the previous literature

	Current (main) manuscript	Avin et al (2005)	Albert and Nelson (2011)	Imai and Yamamoto (2013)
NDE-000<br>NIE_2-000	NPI under set 1	NPI under set 1	NPI under set 1	—<br>—
NDE-111<br>NIE_2-111	NPI under set 1	NPI by symmetry	NPI by symmetry	—<br>—
NIE_1-000	set 1+SP	Not NPI	set 1+SP	set 2
NIE_1-111	set 1+SP	Not NPI by symmetry	set 1+SP by symmetry	set 2
NIE_{12}-000	set 1+SP	Not NPI	set 1+SP	—
NIE_{12}-111	set 1+SP	Not NPI by symmetry	set 1+SP by symmetry	—
NDE-010<br>NDE-101<br>NIE_2-100<br>NIE_2-011	NPI under set 1	—	—	—
NDE-001,-011, -100,-110<br>NIE_1-001,-010, -011,-100,-101,-110<br>NIE_2-001,-010, -101,-110<br>NIE_{12}-001,-010, -011,-100,-101,-110	set 1+SP	—	—	—
SNDE<br>$SNIE_1$<br>$SNIE_2$<br>$SNIE_{12}$	set 1+SP	—	—	—
MS^1-NDE-00<br>MS^1-NDE-11<br>All MS^2 effects	NPI under set 1	—	—	—
Remaining MS^1 effects	set 1+SP	—	—	—
No effect of M_1 on M_2:<br>NDE-00,-01,-10,-11<br>NIE_1-00,-11<br>NIE_2-00,-11	NPI under set 1	—	—	NPI under set 1*
NIE_1-01,-10<br>NIE_2-01,-10	NPI under set 1	—	—	—

Table 3

Descriptive statistics for the Izhevsk Family Study. 1,275 subjects with complete data on Age, SES (first principal component from an asset score analysis), Smoking status and alcohol consumption were included. The number of observations available on the other three variables is listed under n.

	Mean	SD	
Age (yrs)	47.2	10.3	
SES	0.01	1.93	
	Never	Ex	Current
Smoking status	23%	12%	64%
	$\leqslant 10$	$> 10, \leqslant 20$	> 20
Cigarettes per day	40%	46%	14%
	$\leqslant 10l$	$> 10l$	
Ethanol per year	71%	29%	
	Median	IQR	n
BMI (kg/m^2)	25.9	23.0–29.0	800
GGT (IU/l)	30.0	20.0–48.2	798
SBP (mmHg)	142.3	130.0–157.3	813

Table 4

Akaike Information Criterion (AIC) for different associational models for M_1, M_2 and Y. The "basic" model for M_1 includes each of the four confounders ($\mathbf{C}$) and the exposure (X). In addition, the "basic" model for M_2 includes M_1 and the interaction between M_1 and X. The "basic" model for Y additionally includes M_2, the interaction between M_2 and X, the interaction between M_2 and M_1 and the three-way interaction XM_1M_2. "Quad $\mathbf{C}$" denotes that quadratic terms for the two continuous confounders (age and SES) are included. "Inter XC" denotes interaction terms between X and each of the 4 confounders. "Quad M_1" is a quadratic term in M_1 and "quad $\mathbf{M}$" is a quadratic term in both M_1 and M_2. The chosen model, i.e. the model with the highest AIC, is shown in bold type.

Dependent variable	Model	$\frac{1}{2}$AIC
M_1	Basic	283.3
(log BMI)	**Basic + quad C**	**286.6**
	Basic + quad $\mathbf{C}$ + inter XC	285.3
M_2	**Basic**	**−881.4**
(log GGT)	Basic + quad $\mathbf{C}$	−882.7
	Basic + inter XC	−884.9
	Basic + quad M_1	−882.4
Y	Basic	−3468.0
(SBP)	Basic + quad $\mathbf{C}$	−3468.6
	**Basic + quad** M	**−3465.0**
	Basic + quad $\mathbf{M}$ + inter XC	−3468.4

Table 5

The estimated coefficients and SEs for the final chosen associational models.

Model for M_1 (log BMI):		
Variable	Est. Coef.	SE
X	-0.0194	0.013
C_1 (age)	0.0087	0.004
C_2 (SES)	0.0093	0.004
C_3 (smoking status)		
ex-smoker	0.0210	0.023
current-smoker	-0.0569	0.020
C_4 (cigarettes per day)		
10–20	-0.0303	0.017
> 20	0.0189	0.022
C_1^2	-0.0001	0.00004
C_2^2	0.0025	0.001
intercept	3.13	0.089
Model for M_2 (log GGT):		
Variable	Est. Coef.	SE
X	1.75	1.14
M_1	0.79	0.18
$M_1 * X$	-0.41	0.35
C_1	0.01	0.003
C_2	-0.01	0.01
C_3		
ex-smoker	0.02	0.10
current-smoker	0.17	0.09
C_4		
10–20	-0.02	0.08
> 20	-0.11	0.10
intercept	0.46	0.62
Model for Y (SBP):		
Variable	Est. Coef.	SE
X	107.7	142.7
M_1	223.3	97.9
M_2	21.8	25.5
$M_1 * X$	-32.2	44.4
$M_2 * X$	-28.6	36.8
$M_2 * M_1$	-0.994	7.19
$M_2 * M_1 * X$	8.98	11.45
C_1	0.486	0.077
C_2	0.373	0.405
C_3		
ex-smoker	0.575	2.87
current-smoker	4.45	2.50
C_4		
10–20	-1.52	2.16
> 20	-2.20	2.74
M_1^2	-30.7	14.3
M_2^2	-1.62	0.77
intercept	-326.4	176.0
